# An end-to-end pipeline for automated fetal brain segmentation and biometry from 3D SSFP MRI

**DOI:** 10.3389/fnins.2026.1870124

**Published:** 2026-07-09

**Authors:** Yasmin Modarai, Adam Lim, Justin Lo, Matthias W. Wagner, Elka Miller, Logi Vidarsson, Birgit Ertl-Wagner, Dafna Sussman

**Affiliations:** 1 Department of Electrical, Computer and Biomedical Engineering, Faculty of Engineering and Architectural Sciences, Toronto Metropolitan University, Toronto, ON, Canada; 2 Institute for Biomedical Engineering, Science and Technology (iBEST), Toronto Metropolitan University and St. Michael’s Hospital, Toronto, ON, Canada; 3 Division of Neuroradiology, Department of Diagnostic & Interventional Radiology, The Hospital for Sick Children, Toronto, ON, Canada; 4 Institute of Diagnostic and Interventional Neuroradiology, University Hospital Augsburg, Augsburg, Germany; 5 Neurosciences & Mental Health Program, Research Institute, The Hospital for Sick Children, Toronto, ON, Canada; 6 Department of Medical Imaging, University of Toronto, Toronto, ON, Canada; 7 Department of Obstetrics and Gynecology, Faculty of Medicine, University of Toronto, Toronto, ON, Canada

**Keywords:** deep learning, fetal, fetal brain, MRI, nnU-Net, segmentation, SSFP MRI

## Abstract

Fetal magnetic resonance imaging (MRI) plays an essential role for the evaluation of fetal abnormalities, offering improved visualization of developing brain structures and superior soft tissue contrast in comparison to other imaging modalities. Accurate and reproducible assessment of fetal brain biometry is critical for diagnosing neurodevelopmental abnormalities. However, these measurements typically rely on manual segmentation, which is time-consuming, labor-intensive, prone to error and dependent on the interpreting radiologist’s expertise and experience. Recent advancements have enabled automated analysis primarily on Half-Fourier Acquisition Single-shot Turbo spin-Echo (HASTE) sequences, yet these acquisitions are susceptible to inter-slice misalignment and often require time-consuming super-resolution reconstruction. In contrast, 3D Steady-State Free Precession (SSFP) imaging offers smaller slice thickness, improving through-plane resolution, along with reduced motion sensitivity and whole-body coverage in a single scan. In this study, we present an end-to-end deep learning pipeline for automated segmentation and biometry of the fetal brain from whole-body SSFP MRI. The dataset includes manual annotations of the fetal head, brain parenchyma and extraaxial cerebrospinal fluid (CSF). The framework employs nnU-Net for robust head localization and multi-structure segmentation, along with principal component analysis (PCA)-based reorientation and head circumference estimation. A Tri-Attention U-Net architecture was evaluated as a standalone model and within the nnU-Net framework. The final pipeline consists of a Tri-Attention nnU-Net for head localization and nnU-Net for multi-structure segmentation. The pipeline achieved mean Dice similarity coefficients (DSC) of 94.48, 93.58 and 82.75% for the head, brain parenchyma and extraaxial CSF, respectively. These findings demonstrate the feasibility of accurate, fully automated fetal brain biometry from SSFP MRI with potential to reduce inter-observer variability, streamline clinical workflows and enhance clinical decision-making through fast and reproducible quantitative assessment.

## 1 Introduction

Fetal magnetic resonance imaging (MRI) is widely used as a complementary modality to ultrasonography for diagnosing fetal abnormalities (Saleem, 2014). Its use has expanded particularly in the evaluation of the fetal brain due to its superior soft-tissue contrast (Glenn, 2010). Accurate interpretation of fetal MRI is essential for early diagnosis of congenital abnormalities, assessment of brain maturation, and monitoring of neurodevelopment throughout gestation (Glenn, 2010; Griffiths et al., 2019; Hart et al., 2020). Key clinical biometrics, including head circumference, brain parenchyma volume, and extraaxial cerebrospinal fluid (CSF) volume, provide important insights into fetal brain growth and development (Andescavage et al., 2016; Shi et al., 2020). Among these, head circumference is a standard metric for assessing brain growth and serves as a diagnostic marker for conditions such as microcephaly and macrocephaly (Pei et al., 2023). In addition, brain parenchyma volume reflects overall brain maturation and can highlight potential neurodevelopmental abnormalities (Andescavage et al., 2016). Similarly, extraaxial CSF volume can reveal abnormalities such as ventriculomegaly or hydrocephalus, and disproportionate ratios between extraaxial CSF and brain parenchymal volume may indicate additional underlying pathologies (Andescavage et al., 2016).

In clinical practice, obtaining these biometrics typically requires manual segmentation. However, manual segmentation is time-consuming, labour-intensive, prone to error and heavily dependent on the radiologist’s experience and expertise.

Automatic methods for fetal brain segmentation and biometric measurements have been developed for fetal MRI, particularly for Half-Fourier Acquisition Single-shot Turbo Spin-Echo (HASTE)-acquired images (Luis et al., 2025; Matthew et al., 2024; Uus et al., 2023). Several automated fetal brain segmentation models now exist; among them, the recently introduced BOUNTI pipeline provides segmentation of 19 brain regions from 3D fetal MRI (Uus et al., 2023). This capability enables robust and accurate volumetric analysis across a wide range of fetal brain subregions. Building on BOUNTI and the super-resolution reconstruction tool SVRTK (Kuklisova-Murgasova et al., 2012), automated 2D slice-based biometric measurements can be derived from reconstructed 3D T2-weighted HASTE images (Luis et al., 2025). Collectively, these developments represent a shift from traditional manual 2D measurements towards automated, high-resolution 3D deep learning-based analysis in fetal MRI.

Studies have compared the turbo spin-echo-based HASTE sequence to a 3D Steady-State Free Precession (SSFP) sequence for fetal brain MRI (Chung et al., 2000; Griffiths et al., 2013). They found that SSFP can detect most neurodevelopmental abnormalities and often provides better visualization of developmental anatomy in later gestational ages (Chung et al., 2000; Griffiths et al., 2013). Although HASTE sequences are the routine standard for fetal neuroimaging, SSFP can outperform HASTE in certain applications due to reduced sensitivity to inter-slice motion. HASTE sequences acquire individual 2D slices rapidly, making them prone to inter-slice misalignment caused by fetal motion between slices (Machado-Rivas et al., 2023; Manganaro et al., 2023). This limitation necessitates the use of super-resolution reconstruction (SRR) or slice-to-volume (SVR) registration for 3D reconstruction (Griffiths et al., 2013). However, these post-processing methods are computationally expensive, time consuming, and sensitive to image quality, which is often limited in HASTE acquisitions. In comparison, due to their faster acquisition time, 2D multislice SSFP sequences can generate contiguous slices with smaller slice thickness, enabling improved 3D segmentation and volumetric quantification of fetal anatomy without requiring additional post-processing techniques (Griffiths et al., 2013; Machado-Rivas et al., 2023; Manganaro et al., 2023). Furthermore, SSFP permits whole-body fetal imaging in a single acquisition, allowing the same dataset to be used for assessment of the fetus, placenta and amniotic fluid. This provides a unified dataset that captures all relevant fetal anatomy, potentially eliminating the need for multiple sequences and thereby reducing overall scan time. In addition, using a single acquisition ensures consistent spatial alignment across organs, improving the accuracy of multi-organ segmentation and biometric extraction. Taken together, SSFP offers a more efficient and comprehensive framework for fetal assessment compared with HASTE-based workflows.

Current automated biometric pipelines rely on HASTE images combined with SRR to approximate 3D structure. In contrast, whole-body SSFP imaging provides improved through-plane resolution which results in more direct and accurate volumetric and structural segmentation without requiring any additional post-processing algorithms. To our knowledge, this is the first study to utilize whole-body SSFP MRI sequences for automated fetal brain biometry, integrating head localization, true axial-plane standardization and multi-structure segmentation of brain parenchyma and extraaxial CSF within a unified pipeline.

## 2 Materials and methods

### 2.1 Dataset collection

**Figure 1:**
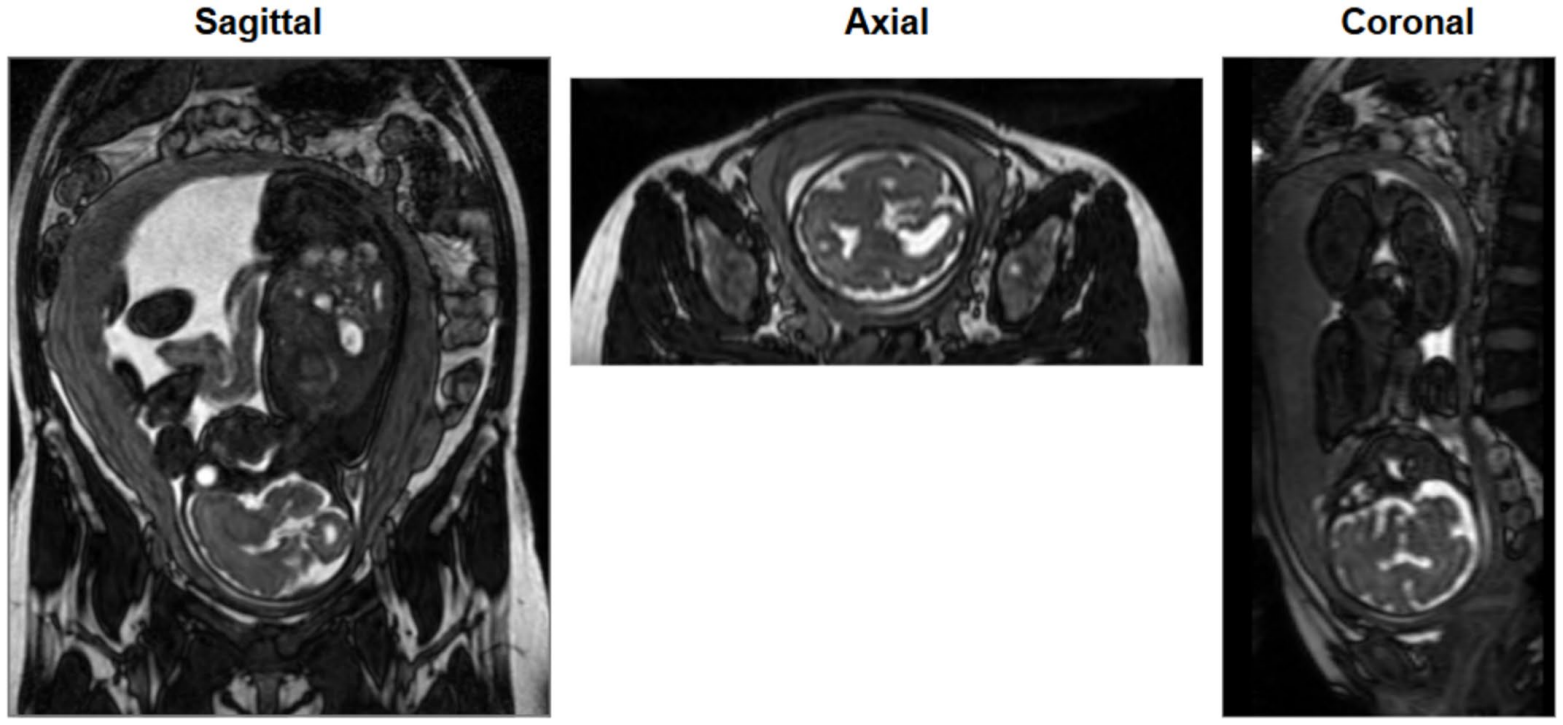
Example of SSFP whole-body fetal MRI in sagittal, axial, and coronal planes.

The dataset consisted of 84 de-identified 3D whole-body fetal MRIs acquired using a 3D Steady-State Free Precession (SSFP) sequence with sensitivity encoding (SENSE) in two directions on a Prisma Fit 3.0 T MRI scanner (Siemens Healthineers, Erlangen, Germany). These T2-weighted images were acquired coronally relative to the mother with the following parameters: repetition time (TR) = 6.5 ms, echo time (TE) = 2.99 ms, voxel size = 0.78×0.78×2 mm^3,^ flip angle = 50°, acquisition matrix size = 512×512 and slice thickness = 2 mm. The acquisition time per scan was 29 s. Data was obtained from The Hospital for Sick Children in Toronto, Canada (REB# 1000062640), and all processing and analysis were conducted at Toronto Metropolitan University (REB# 2018–398). An example of a dataset is shown in Figure 1.

**Figure 2:**
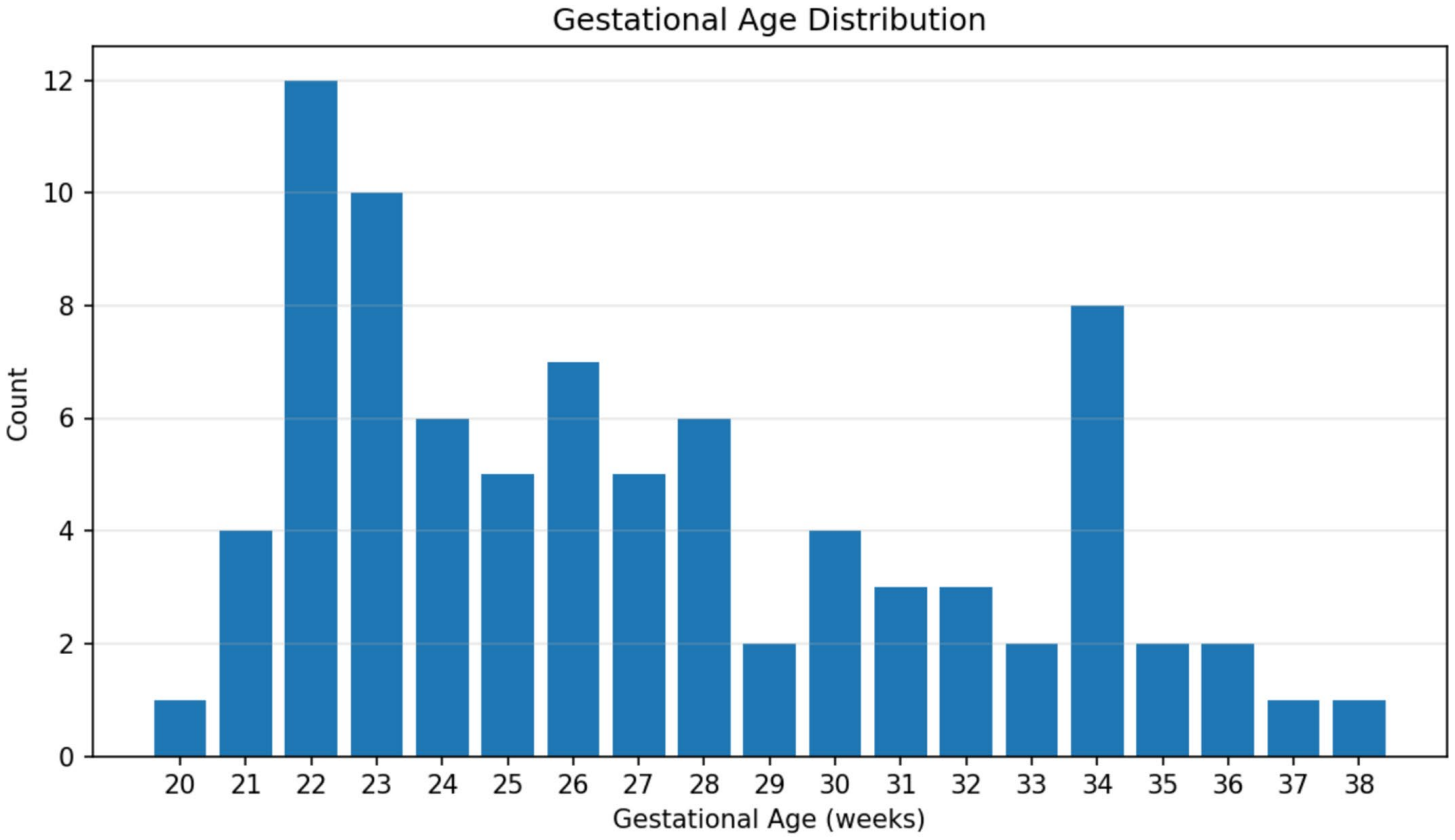
Histogram of gestational age distribution used in our study.

The study cohort included 84 fetal MRIs acquired between 20 and 34 weeks of gestation (histogram distribution shown in Figure 2) where 32 cases represented structurally normal fetuses. The remaining 52 cases included fetuses with enlarged extraaxial CSF spaces and ventriculomegaly (*n* = 5), enlarged extraaxial CSF spaces and colpocephaly (*n* = 2), only enlarged extraaxial CSF spaces (*n* = 12), only ventriculomegaly (*n* = 23) and only colpocephaly (*n* = 10). Scans with minor abnormalities, such as mild ventriculomegaly were included, while those with rare brain abnormalities, twin pregnancies, or severe artifacts were excluded. Minor imaging artifacts, including mild motion, chemical shift or radiofrequency distortion were permitted provided they did not substantially affect the visibility of the cranial anatomy.

Manual segmentation of the head, brain parenchyma and extraaxial CSF was performed by a single annotator in-house using Amira Software (Thermo Fisher Scientific, MA, United States). Each case was segmented on a slice-by-slice basis in the sagittal plane with reference to the axial and coronal views for boundary confirmation. Mean segmentation time was approximately 4 h per volume. All segmentation masks were subsequently validated by an expert fetal radiologist with over 20 years of experience. In cases where the reviewing radiologist identified boundary errors, the annotator corrected the mask and resubmitted it for subsequent approval. Inter-rater reliability could not be formally assessed due to the availability of only one trained annotator; however, the structured review-and-correction protocol was designed to minimize annotation error.

### 2.2 Dataset preprocessing

Original 3D volumes were resized to uniform dimensions of 256x256x64. To mitigate extreme outliers, voxel intensities were clipped at the 99.5th percentile and subsequently normalized to the range of [0, 1]. The corresponding ground truth head segmentation masks were resized to the same dimensions. For deep supervision, the ground truth head segmentation masks were additionally down sampled to match the resolution of each decoder output required by the model. For the training set, data augmentation techniques were applied including random rotations, random brightness and contrast adjustments to increase variability and improve model generalization. The preprocessing for the multi-structure brain segmentation model was identical, except that the cropped 3D volumes were resized to a uniform resolution of 128x128x64. This preprocessing strategy was applied consistently across all experiments, with the exception of the nnU-Net models, which employed their own preprocessing pipeline. All experiments were evaluated using 5-fold cross validation, with random patient-level splitting to prevent data leakage between the training and validation sets. Each split consisted of 68 training volumes and 16 validation volumes. To ensure a fair comparison, the same fold splits generated by nnU-Net were used across all experiments.

### 2.3 Pipeline overview

**Figure 3:**
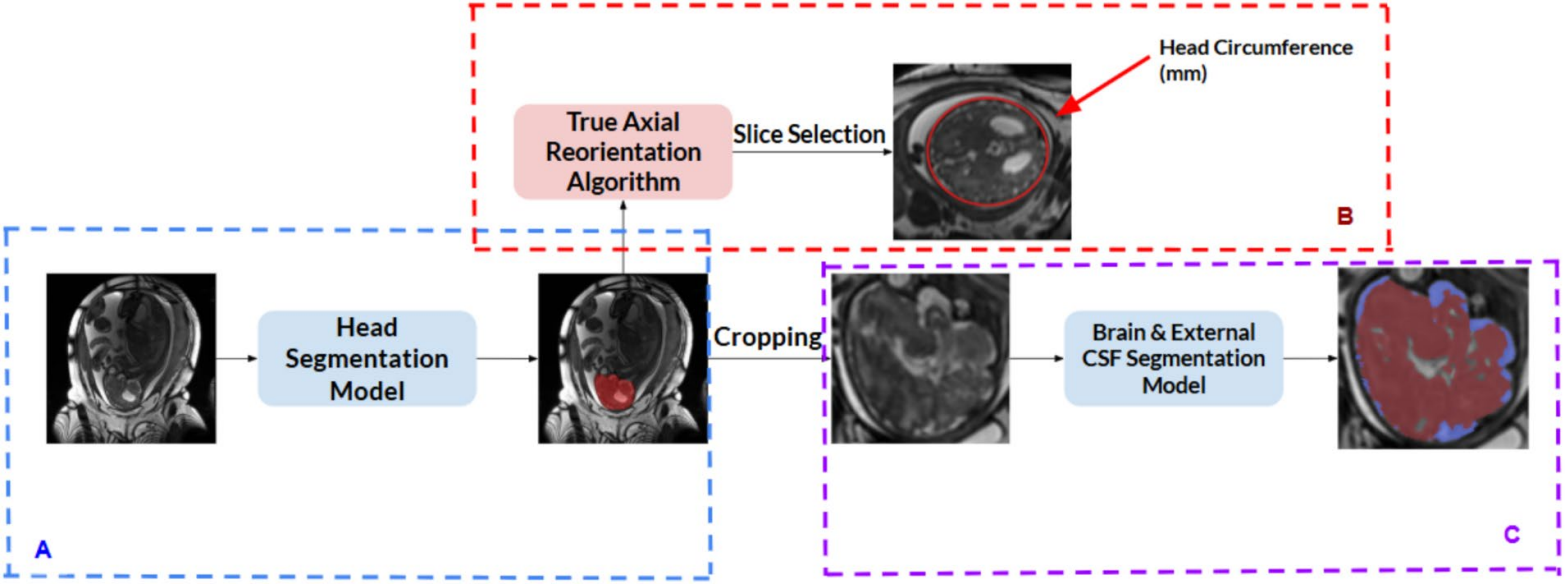
Overview of the proposed pipeline. **(A)** First, the SSFP whole-body MRI volumes are provided as input to the head segmentation model. This model is used as a preprocessing step to localize the head region. **(B)** The resulting head segmentation masks are also used to determine the true axial plane relative to the brain using a PCA-based algorithm. Head circumference is computed from the optimal axial slice using an automatic ellipse-fitting algorithm. **(C)** The cropped volumes are used as input to the multi-structure segmentation model to delineate the brain parenchyma (shown in red) and extraaxial cerebrospinal fluid (shown in blue).

The end-to-end pipeline consisted of three parts: (1) a head segmentation model for extraction of the fetal head region, (2) a true axial reorientation algorithm for standardization to the axial plane and derivation of head circumference measurements, and (3) a brain segmentation model for delineation of brain parenchyma and extraaxial CSF to enable volumetric biometry extraction. This is illustrated in Figure 3.

### 2.4 Proposed model architecture

**Figure 4:**
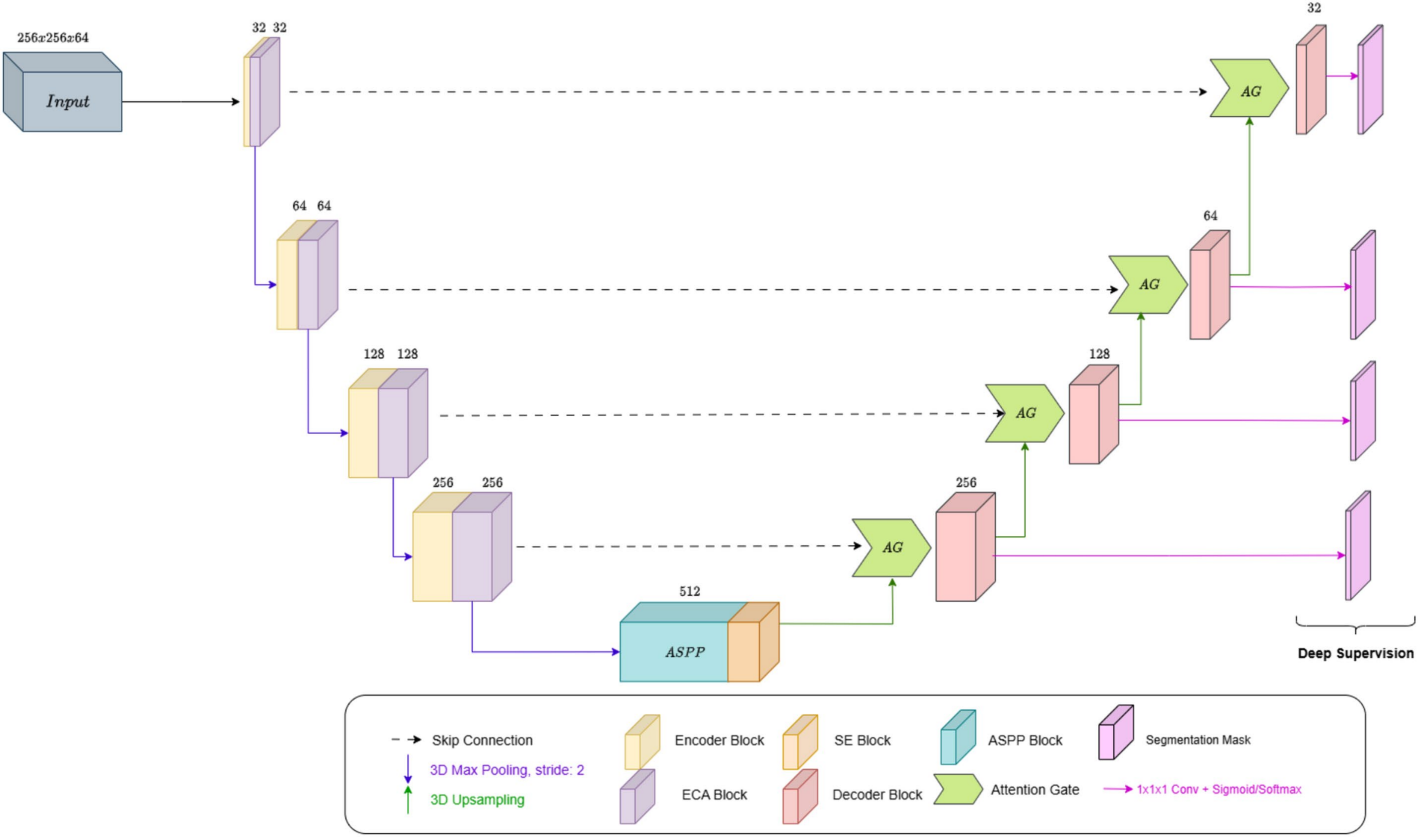
Schematic of the tri-attention 3D U-net architecture. Input is the 3D MRI volume (resized to 256x256x64). The number of channels is displayed at the top of each block. The structure of this model follows the U-Net encoder-decoder structure. The encoder block is denoted by the yellow block which is followed by the ECA block (purple). The bottleneck block consists of the ASPP block (blue) and a SE block (orange). Attention gate blocks are represented by green blocks with “AG.” In the decoder pathway, red blocks represent the decoder blocks, while pink blocks denote the segmentation outputs generated at each decoder stage for deep supervision.

The proposed model is a 3D U-Net-based architecture with multi-stage attention mechanisms and deep supervision for volumetric whole-body fetal MRI segmentation. The encoder consists of residual-style convolutional blocks with an additional Efficient Channel Attention (ECA) mechanism. ECA in the encoder block allows for a computationally efficient method to capture channel dependencies within a large feature map (Wang et al., 2020). The bottleneck incorporates an Atrous Spatial Pyramid Pooling (ASPP) block combined with a Squeeze-and-Excitation (SE) block to capture multi-scale contextual information while globally reweighting channel features (Chen et al., 2016; Chowdhury and Mirzaei, 2025; Hu et al., 2017). Integrating ASPP and SE at the bottleneck allows the model to extract more abstract, high-level feature representations. The decoder consists of a gated attention mechanism to focus on relevant regions of the encoder feature maps (Oktay et al., 2018). Lastly, deep supervision is applied at each decoder stage to enhance gradient flow and improve convergence (Le’Clerc Arrastia et al., 2021). Overall, the combination of ECA, SE, ASPP and attention gates in this architecture allows the model to effectively and efficiently capture rich contextual and spatial information for accurate 3D segmentation. This architecture was used for both the head segmentation model and the multi-structure brain segmentation model which is shown in Figure 4.

Although this architectural configuration is not novel, it is specifically suited to the characteristics of whole-body fetal MRIs. Each component addresses specific challenges associated with our dataset. Firstly, the ECA block in the encoder allows the model to detect subtle intensity variations commonly observed in fetal MRIs. The combination of ASPP and SE modules help capture contextual information across multiple resolutions and scales, which is critical for handling large variability in organ size and shape across gestational ages. Furthermore, attention gates in the decoder suppress irrelevant background regions, which is particularly beneficial in the presence of motion artifacts, and when the fetal head occupies a small portion of the field of view. Lastly, deep supervision improves gradient propagation and stabilizes training, which is advantageous given the limited dataset size and high variability in fetal MRIs.

### 2.5 Head circumference algorithm

Accurate measurement of fetal head circumference from SSFP MR images requires compensating for the fact that image acquisition is performed in the maternal reference frame, resulting in variable fetal head orientations. Without correcting for this misalignment, standard anatomical planes (axial, sagittal and coronal) contain no correspondence to fetal brain anatomy, leading to inconsistent or incorrect biometric measurements. The proposed algorithm addresses this by utilizing the output segmentations of the fetal head to standardize orientation and identifies the optimal slice for fetal head circumference measurement. The pipeline consists of three stages: (1) principal component analysis (PCA)-based reorientation, (2) optimal slice selection, and (3) elliptical fitting.

#### 2.5.1 PCA-based reorientation

**Figure 5:**
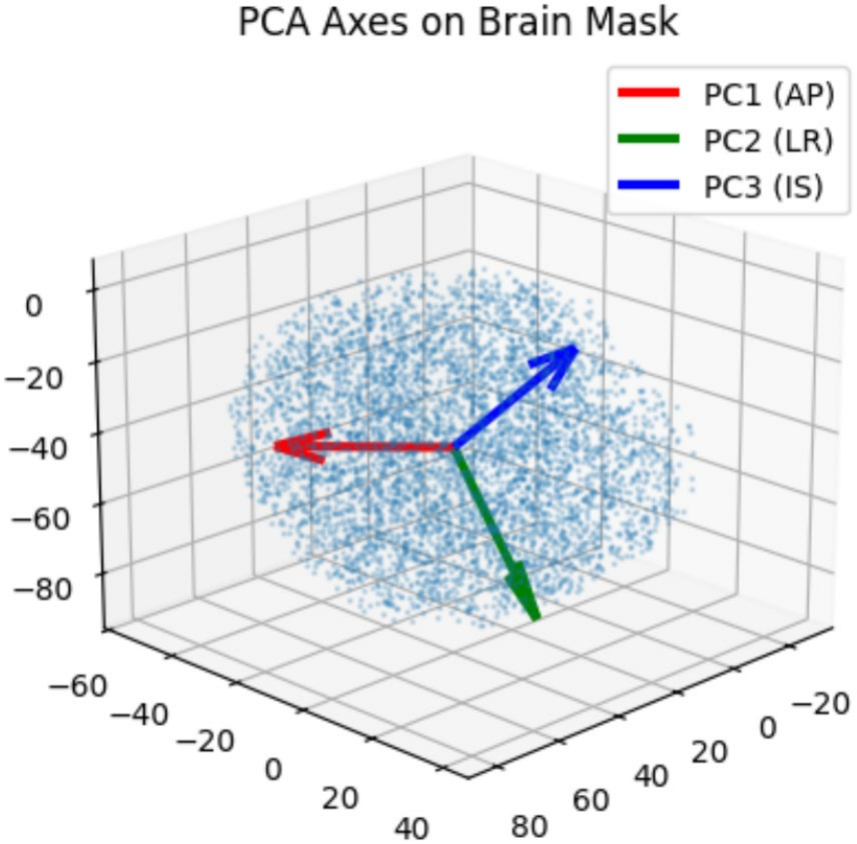
Example of PCA applied to the point cloud representing the fetal head. PC1 (red) represents anteroposterior axis, PC2 (green) represents left–right axis, and PC3 (blue) represents inferior–superior axis.

The segmentation volume is first converted into a point cloud by extracting the voxel coordinates belonging to the fetal head (shown in Figure 5). PCA is then performed on this point set to determine the directions of maximal variance, which correspond to the anatomical long, intermediate, and short axes of the fetal brain. The first principal component (largest variance) approximates the anteroposterior (front to back) axis, while the second and third components capture the left to right and superior to inferior axes, respectively. These components are used to construct a rotation matrix to reorient the volume, such that the axial plane corresponds to slices perpendicular to the superior–inferior axis of the brain. This step standardizes the anatomical orientation across subjects by eliminating variability due to fetal presentation and maternal acquisition planes, resulting in a consistent coordinate system for subsequent analysis.

#### 2.5.2 Slice selection

After reorienting the masks, each axial slice is analyzed by computing the cross-sectional area of the fetal head, which is defined as the number of voxels belonging to the head segmentation within that slice. The slice with the maximum area is selected, as this typically corresponds to the level of the thalami and cavum septi pellucidi, which defines the standard transventricular plane used for head circumference measurements in ultrasound, and approximately the same level used in MRI, corresponding to the maximum biparietal diameter at the level of the temporal lobes and atria of the lateral ventricles (Poojari et al., 2022).

#### 2.5.3 Elliptical fitting

From the selected axial slice, the outer boundary is extracted from the segmentation mask. An ellipse is then fitted to this boundary using a least-squares optimization approach. The fitted ellipse is parameterized by its major axis (the longest diameter), minor axis (the shortest diameter), and orientation, which together approximate the shape of the skull. The head circumference is calculated from these axes using Ramanujan’s approximation, which provides an accurate estimate of an ellipse’s perimeter (denoted as *p* in Equation 2). Equations 1, 2 display Ramanujan’s approximation where *a* is denoted as the major axis, *b* is the minor axis of the ellipse and *p* is the circumference. This approach reduces sensitivity to minor segmentation irregularities and produces a reliable, reproducible estimate of head circumference, closely matching clinical ultrasound-based measurements.


$$h=\frac{{\left(a-b\right)}^{2}}{{\left(a+b\right)}^{2}}$$ (1)

$$p\approx \pi (a+b)(1+\frac{3h}{10+\sqrt{4-3h}})$$ (2)

### 2.6 Implementation, experiments and evaluation metrics

The model was trained and validated on a NVIDIA GeForce RTX 4090 (24GB) GPU using PyTorch and MONAI (Cardoso et al., 2022) for preprocessing and the implementation of baseline architectures. The training spanned a total of 100 epochs with a batch size of 1. Early stopping was implemented with a patience of 15 epochs based on the validation Dice similarity coefficient (DSC) to prevent overfitting. The built-in Adam optimizer was implemented with a learning rate of 0.001. The evaluation metrics used are DSC, intersection-over-union (IoU), sensitivity, specificity, precision and 95% Hausdorff distance (HD95). DSC and IoU measure the degree of overlap between the ground truth and predicted segmentation masks. DSC is generally more tolerant of over- and under-segmentation errors compared to IoU (Müller et al., 2022). Sensitivity measures the model’s ability to correctly identify positive voxels, whereas specificity measures the model’s ability to correctly identify negative voxels out of all negatively labelled voxels. The Equations 3–7 show each evaluation metric where *TP* is True Positive, *TN* is True Negative, *FN* is False Negative and *FP* is False Positive.


$$\mathrm{DSC}=\frac{2\mathrm{TP}}{\mathrm{FP}+2\mathrm{TP}+\mathrm{FN}}$$ (3)

$$\mathrm{IoU}=\frac{\mathrm{TP}}{\mathrm{TP}+\mathrm{FN}+\mathrm{FP}}$$ (4)

$$\text{Sensitivity}=\frac{\mathrm{TP}}{\mathrm{TP}+\mathrm{FN}}$$ (5)

$$\text{Specificity}=\frac{\mathrm{TN}}{\mathrm{TN}+\mathrm{FN}}$$ (6)

$$\text{Precision}=\frac{\mathrm{TP}}{\mathrm{TP}+\mathrm{FP}}$$ (7)

HD95 is commonly used to measure the similarity of boundaries between the ground truth and predicted pixels. Equation 8 defines the HD95 metric, where *d_PG_* and *d_GP_* represent the 95th percentile directed distances from the predicted segmentation (*P*) to the ground truth segmentation (*G*) and vice versa, respectively. HD95 is calculated as the maximum of these two values.


$$\mathrm{HD}95(P,G)=\max\{d_{\mathrm{PG}},d_{\mathrm{GP}}\}$$ (8)

The loss function utilized was a combination of focal loss and DSC loss (Yeung et al., 2022). The DSC loss (shown in Equation 9) is commonly used for segmentation tasks as it directly optimizes the overlap between predictions and ground truth masks. Focal loss is a modified cross-entropy loss function that measures the difference between the predicted and ground truth segmentation probabilities while emphasizing hard-to-classify examples by down-weighting well classified predictions. This is achieved through the factor ${\left(1-p\right)}^{\gamma }$ , where *p* denotes the predicted probability for the ground truth class and γ>0 controls the degree of focussing (Lin et al., 2017). As γ increases, the contribution of easily classified examples is reduced, allowing the model to focus on more difficult examples. The Focal loss equation is shown in Equation 10, where $p_{\mathrm{ic}}$ stands for the probability of voxel *i* belonging to class *c*. For the multi-structure segmentation model, a combination of Dice loss and focal loss was used.


Dice Loss=1−DSC (9)

$$\text{Focal Loss}(p_{\mathrm{ic}})=-{\left(1-p\right)}^{\gamma }\log(p_{\mathrm{ic}})$$ (10)

The proposed Tri-Attention 3D U-Net architecture was evaluated against other widely used 3D architectures, including the original U-Net (Ronneberger et al., 2015), Attention U-Net (Oktay et al., 2018), SegResNet (Myronenko, 2018) and Dynamic U-Net (Yang et al., 2024). These comparisons were performed for both the head segmentation and multi-structure segmentation tasks. All models were trained and evaluated with the same preprocessing and training setup to ensure fair comparisons.

In addition, the head segmentation model was evaluated against other established brain extraction methods such as HD-BET (Isensee et al., 2019) and FSL’s Brain Extraction Tool v2 (BET2) (Smith, 2002).

An ablation study was conducted to observe the impact of each component in the architecture on the head segmentation dataset. The ablation study involved the addition/removal of each attention component and the ASPP block in the bottleneck. All experiments were performed using deep supervision and the same hyperparameters to ensure consistency.

To further benchmark the performance of the proposed Tri-Attention U-Net architecture, we conducted a separate evaluation using nnU-Net. This state-of-the-art segmentation framework automatically configures the architecture and training pipeline to the given dataset (Isensee et al., 2021). Due to its self-adapting nature, nnU-Net does not share the same fixed architecture or training protocol as our previous experiments and therefore cannot be directly compared under identical conditions. Instead, nnU-Net was evaluated in a separate experiment using its default configuration, and its performance was compared with the proposed Tri-Attention U-Net. This comparison provides a practical benchmark against a state-of-the-art segmentation pipeline under its optimized settings.

Brain parenchyma and extraaxial CSF volumes were computed from the predicted segmentation masks using voxel-wise volumetric calculations. To ensure accurate and reproducible volumetric measurements, both predicted and ground truth masks were first resampled back to the original image space prior to volume comparison. The physical volume of each structure was calculated using the voxel spacing derived from the image affine matrix. The Equation 11 is shown below where *d_x_*, *d_y_* and *d_z_* represent the voxel dimensions (in millimetres) along each spatial axis. It is then divided by 10^6^ to convert the volume from cubic millimetres to litres.


$$\text{Volume}(L)=\frac{\left(\text{Number of voxels})(d_{x}\cdot d_{y}\cdot d_{z}\right)}{10^{6}}$$ (11)

Volumetric difference, as seen in Equation 12 was determined by using the absolute percentage volume difference between predicted (*V_P_*) and ground truth segmentations (*V_GT_*) for each structure (brain parenchyma and extraaxial CSF).


$$\text{Volumetric Difference}=|\frac{V_{P}-V_{\mathrm{GT}}}{V_{\mathrm{GT}}}|\times 100\%$$ (12)

## 3 Experimental results and discussion

### 3.1 Head segmentation model results

Table 1 displays the segmentation evaluation metrics (DSC, IoU, sensitivity, specificity, precision and HD95) for the proposed model architecture and baseline architectures. These results were conducted using the head segmentation dataset. Normality of the data distribution was assessed using the Shapiro–Wilk test prior to significance testing. When assumptions of normality were met, a paired t-test was conducted to assess statistical significance between the proposed model and each baseline across the cross validation folds. Statistical significance is indicated by a ‘*’ (*p* < 0.0125), based on Bonferroni correction ( $p=\frac{\text{original}\;p-\mathrm{val}\;(0.05)}{\text{Number of comparisons}\;(4)}=0.0125)$ for multiple comparisons. Statistical testing was conducted independently for each metric using paired observations from identical validation folds to preserve correspondence between models during cross validation evaluation.

**Table 1:** 5-fold cross validation results for comparative analysis on head segmentation dataset.

Model	DSC	Iou	Sensitivity	Specificity	Precision	hd95
U-Net	80.43% ± 2.86	68.79% ± 3.59	94.88% ± 2.41	99.64% ± 0.09	72.03% ± 5.63	24.15 ± 7.45
Attention U-Net	82.31% ± 4.25	71.32% ± 5.37	95.47% ± 2.71	99.69% ± 0.10	75.04% ± 7.09	23.72 ± 14.07
Segresnet	77.73% ± 5.40*	64.68% ± 6.80*	95.88% ± 2.63	99.56% ± 0.09*	68.22% ± 5.99*	**9.30 ± 3.07**
Dynamic U-Net	82.51% ± 5.19	71.52% ± 6.92	96.11% ± 1.88	99.68% ± 0.12	74.76% ± 6.92	14.71 ± 1.45
Tri-Attention U-Net	**85.70% ± 3.38**	**75.79% ± 4.67**	**97.98% ± 0.53**	**99.74% ± 0.05**	**78.65% ± 4.05**	13.32 ± 7.67

The best results are shown in bold. A table including specific *p*-values, 95% confidence interval differences and Cohen’s d effect sizes can be viewed in Supplementary Table 1.

The proposed Tri-Attention U-Net achieved the highest overall performance with a DSC of 85.70% ± 3.38 and IoU of 75.79% ± 4.67. Furthermore, it demonstrated improved sensitivity, precision and specificity compared to the other networks, indicating strong detection capabilities. The improvement in sensitivity suggests that the combined ECA and attention gate mechanisms better recover low-contrast head boundaries, reducing missed detections. This is clinically consequential as false negatives in the head localization stage shift the region of interest and corrupt downstream biometry measurements, including head circumference.

SegResNet demonstrated statistically significant differences in several metrics (IoU, specificity and precision), but overall performance remained lower than that of the proposed model. Notably, SegResNet achieved the lowest HD95 among the evaluated models, indicating improved boundary consistency. This can be attributed to its residual architecture, which promotes smoother and more stable predictions with fewer boundary outliers. In contrast, the proposed Tri-Attention U-Net recovers greater segmentation extent through multi-scale context from the ASPP and skip-level attention, improving DSC but occasionally introducing boundary perturbations. For clinical biometry, where head circumference is derived from the head segmentation mask, a model with lower HD95 may yield more repeatable circumference estimates even when the volumetric overlap is lower.

These results demonstrate the effectiveness of the proposed architecture for improving segmentation accuracy in the head segmentation task for whole-body SSFP fetal MRIs.

**Figure 6:**
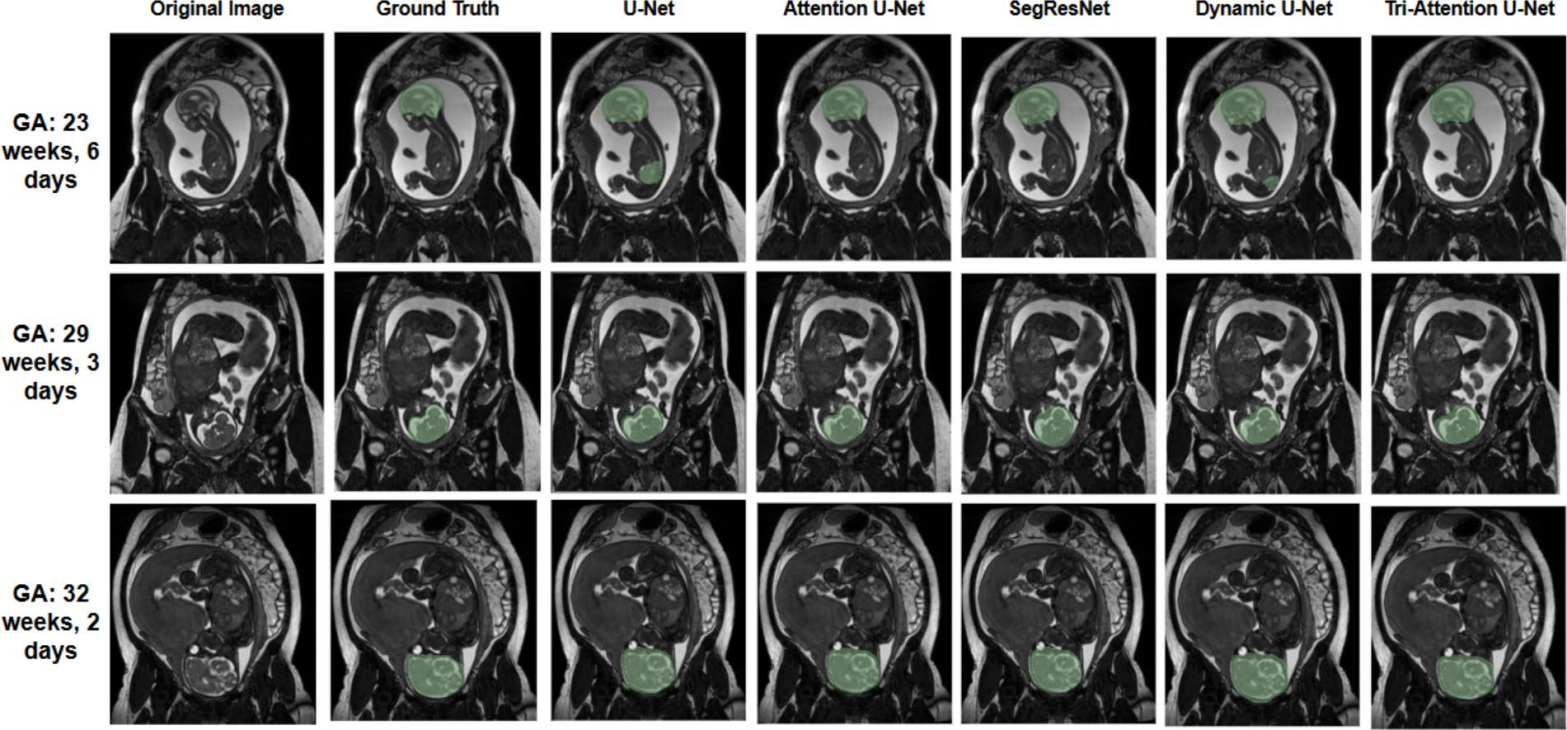
Example outputs from each network outlined in Table 1. The first column displays the whole-body MR image input. The second column displays the corresponding ground truth segmentation. The following columns display prediction segmentation masks for U-Net, Attention U-Net, SegResNet, Dynamic U-Net and the proposed Tri-Attention U-Net, respectively. The highlighted green area represents the head segmentation mask.

As shown in Figure 6, the networks perform relatively well on later gestational ages. In the 23-week example, the U-Net and Dynamic U-Net incorrectly segment regions corresponding to the fetal abdomen or posterior body as part of the head. This limitation is particularly important as the head segmentation serves as a critical preprocessing step for subsequent stages of the pipeline. Specifically, it is used to localize and crop the head region, as well as compute head circumference measurements. Minor segmentation errors at this stage can propagate through the pipeline, potentially affecting the accuracy of downstream analyses.

The proposed Tri-Attention U-Net model outperformed other established brain extraction methods like HD-BET (Isensee et al., 2019) and BET2 (Smith, 2002) with results shown in Table 2. This performance difference is expected, as these methods are primarily developed and trained on pediatric and adult brain MRIs, which differ substantially from whole-body fetal MRIs in terms of anatomical scale, tissue contrast, motion artifacts and the presence of both maternal and fetal structures that complicate accurate head localization.

**Table 2:** Results of comparative analysis on established brain extraction methods compared to the proposed model.

Brain extraction method	DSC	Iou	Sensitivity	Specificity	Precision	hd95
BET2	4.89% ± 3.91	2.55% ± 2.09	88.23% ± 30.25	63.80% ± 19.38	2.56% ± 2.09	220.02 ± 62.66
HD-bet	17.86% ± 11.05	10.22% ± 6.81	51.47% ± 32.08	96.58% ± 2.05	11.11% ± 7.06	207.91 ± 29.92
Tri-Attention U-Net	**85.70% ± 3.38**	**75.79% ± 4.67**	**97.98% ± 0.53**	**99.74% ± 0.05**	**78.65% ± 4.05**	13.32 ± 7.67

Best results are shown in bold.

**Figure 7:**
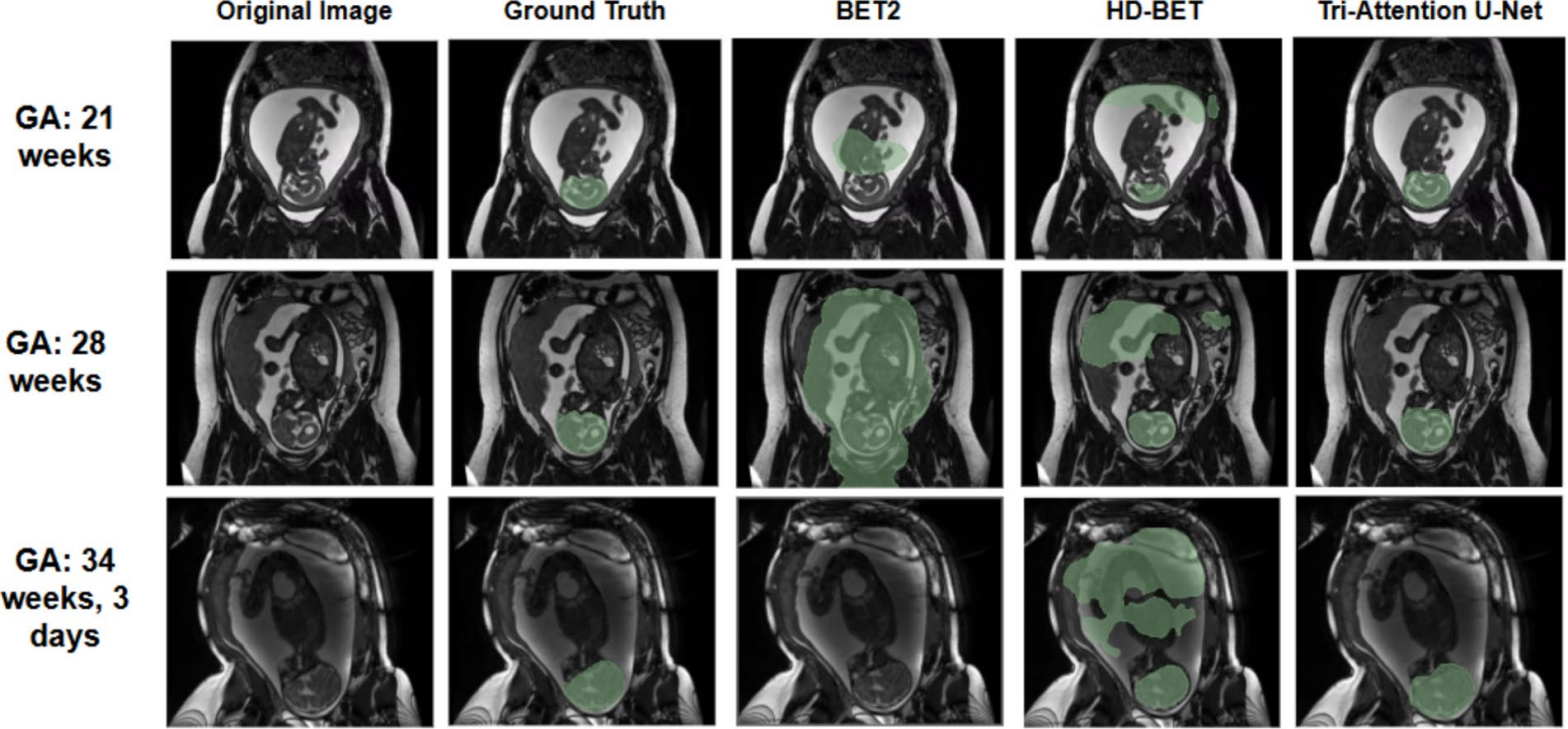
Example outputs from each network outlined in Table 2. The first column displays the whole-body MR image input. The second column displays the corresponding ground truth segmentation. The following columns display prediction segmentation masks for BET2, HD-BET and the proposed Tri-Attention U-Net, respectively. The highlighted green area represents the head segmentation mask.

BET2 performs significantly worse than HD-BET as seen in Figure 7. It frequently fails to detect the head region or severely over-segments, resulting in large portions of the fetus being incorrectly classified as brain tissue. This behaviour reflects the fundamental domain mismatch between adult brain extraction and whole-body fetal MRI. BET2 relies on intensity-based skull-stripping heuristics calibrated for adult cranial proportions, which do not transfer to the fetal context where the skull is completely ossified and surrounded by maternal tissue. At 34 weeks, BET2 produced no valid segmentation in Figure 7, a critical failure for any biometry pipeline.

In comparison, HD-BET demonstrates improved localization of the head region. However, it still over-segments and incorrectly labels non-cranial regions as part of the head. While HD-BET provides better coverage than BET2, its predictions remain unreliable due to the inclusion of substantial false positive regions.

Overall, these results demonstrate that conventional brain extraction tools do not generalize well to fetal MRI applications. The superior performance of the proposed Tri-Attention U-Net highlights the importance of designing models specifically tailored to the unique challenges of fetal whole-body MRIs.

### 3.2 Ablation study

An ablation study, shown in Table 3, was performed to determine which components in the architecture had the most impact. Prior to statistical testing, normality of each metric distribution was assessed per variant using the Shapiro–Wilk test. All distributions satisfied the normality assumption, besides specificity of the baseline U-Net.

**Table 3:** 5-fold cross validation results for the head segmentation ablation study.

Model	DSC	Iou	Sensitivity	Specificity	Precision	hd95
baseline U-Net	80.43% ± 2.86	68.79% ± 3.59	94.88% ± 2.41	99.64% ± 0.09	72.03% ± 5.63	24.15 ± 7.45
ECA only	74.47% ± 3.22*	60.89% ± 3.83*	96.02% ± 1.92	99.46% ± 0.07*	63.65% ± 3.55*	16.20 ± 4.21
ASPP only	72.02% ± 5.16*	57.91% ± 6.55*	95.73% ± 1.94	99.39% ± 0.15*	60.69% ± 7.24*	19.29 ± 4.15
Attention gate only	75.87% ± 4.24	62.61% ± 13.45	95.73% ± 1.92	99.51% ± 0.11	65.98% ± 5.84	15.20 ± 4.21
SE only	77.72% ± 2.84*	64.88% ± 3.40*	95.98% ± 2.10	99.56% ± 0.07*	67.99% ± 4.21*	**11.25 ± 5.82**
Tri-attention U-Net	**85.70% ± 3.38**	**75.79% ± 4.67**	**97.98% ± 0.53**	**99.74% ± 0.05**	**78.65% ± 4.05**	13.32 ± 7.67

The best results are shown in bold. Pairwise comparisons were conducted between the full Tri-Attention U-Net and each ablation variant where “*” indicates statistical significance (*p* < 0.01 due to 5 comparisons with Bonferroni-correction). A table including specific *p*-values, 95% confidence interval differences and Cohen’s d effect sizes can be viewed in Supplementary Table 2.

The full Tri-Attention U-Net achieved the highest Dice score (85.70% ± 3.38) and IoU (75.79% ± 4.67), with statistically significant improvements over the baseline model and all ablation variants for both metrics. No individual component alone approached the full-model performance, confirming that the ECA, ASPP, attention gate and SE block contributed complementary rather than redundant functions.

Among the ablation variants, the attention gate achieved the highest DSC (75.87%), indicating it is the strongest individual contributor to spatial localization. ECA-only and ASPP-only performed below the baseline U-Net. In isolation, ECA amplifies false positive activations in non-head regions where channel statistics alone cannot distinguish the head from the body, which explains the reduction in precision relative to the baseline U-Net.

HD95 differences were not statistically significant for any comparison. Attention-based mechanisms improved DSC by suppressing non-head activations across coarse spatial scales, but they do not sharpen local boundary transitions, which is later seen in the multi-structure segmentation task. The SE variant achieved the lowest mean HD95 (11.25 mm) but with the widest confidence interval ([3.17, 19.33]), indicating boundary instability across folds.

The full model’s improvements in sensitivity (97.98%) and precision (78.65%) are clinically significant for the head localization task. Because the head segmentation serves as the basis for head circumference measurement, uncontrolled false positives can inflate the estimated boundary contour and adversely affect biometric accuracy.

### 3.3 Head circumference algorithm results

**Figure 8:**
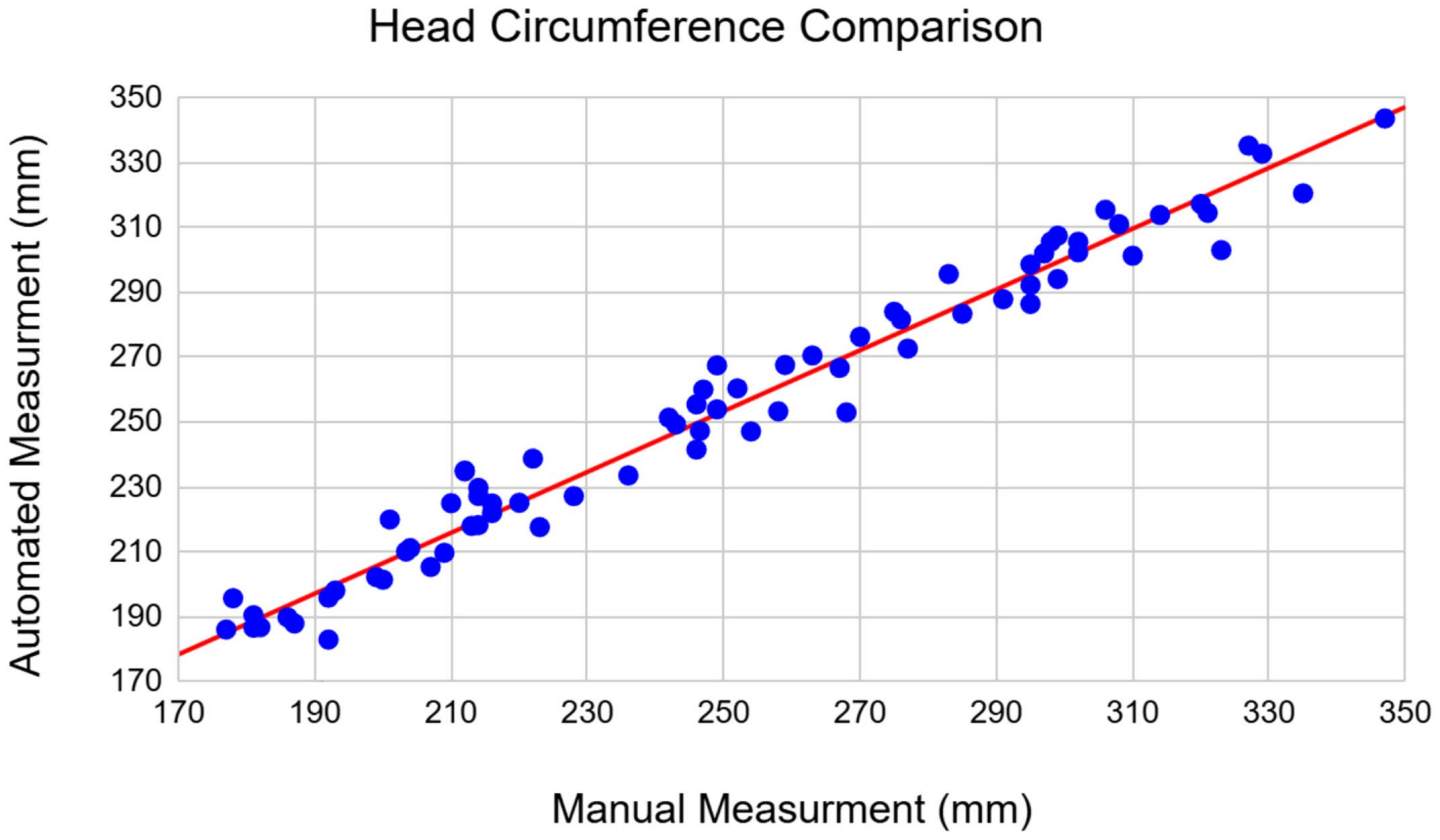
Correlation between manual and automated head circumference measurements. A strong linear correlation was observed (r = 0.982), indicating high agreement between the automated and manual measurements. The automated head circumference measurements were robust across the full gestational age range of the dataset, displaying no measurable drop in performance.

**Figure 9:**
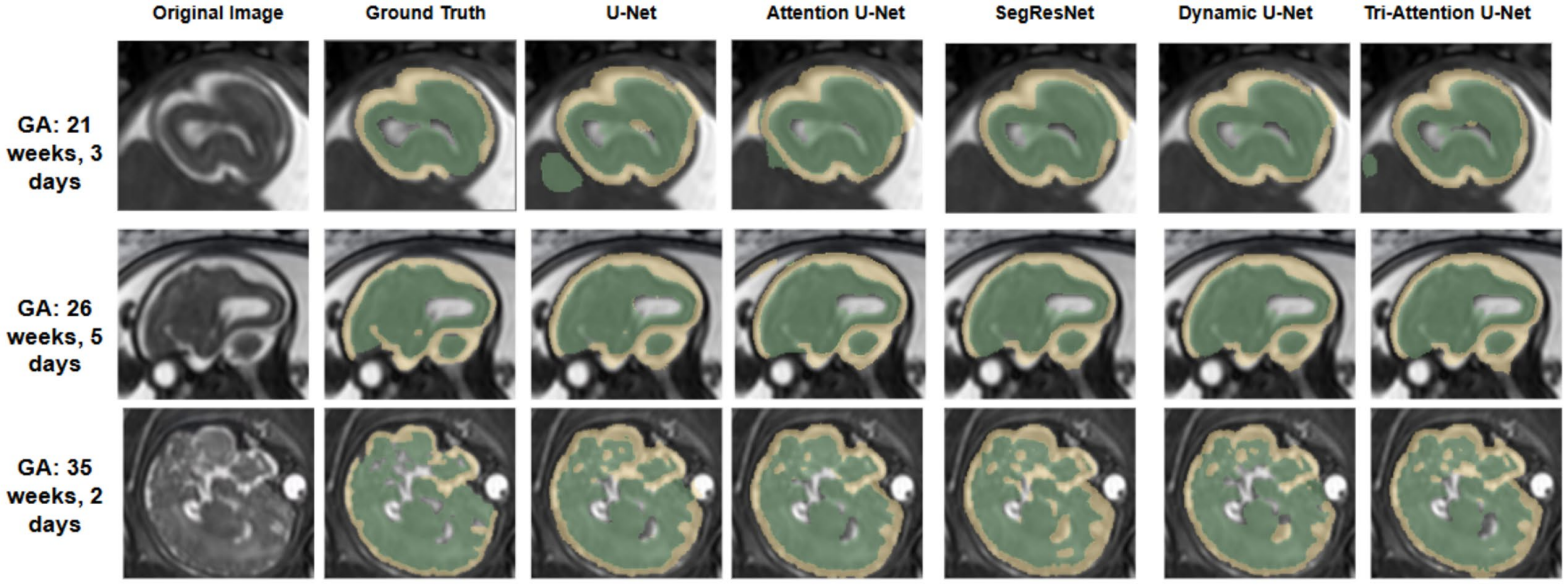
Example outputs from each network outlined in Tables 4, 5. The first column displays the cropped head MR input. The second column displays the corresponding ground truth segmentation overlaid on the input. The following columns display prediction segmentation masks for U-Net, Attention U-Net, SegResNet, Dynamic U-Net and Tri-Attention U-Net, respectively. The green and yellow highlights represent the brain parenchyma and extraaxial CSF, respectively.

Automated fetal head circumference measurements were evaluated against manual annotations across *n* = 84 datasets. Ground truth measurements were extracted from the radiology report. The algorithm achieved a mean absolute error of 3.24% which equated to a physical average error of 7.62 mm. Pearson correlation analysis demonstrated a strong linear relationship between the predicted and manual measurements (r = 0.982). This agreement is further supported in the scatter plot shown in Figure 8, where the data points are situated close to the trend line, indicating minimal deviation between predicted and reference measurements. Bland–Altman analysis demonstrated a mean bias of 3.40 mm with 95% limits of agreement of approximately ±17.63 mm (−14.22 mm to +21.03 mm), corresponding to approximately ±7.1% relative agreement. This indicates a small systematic overestimation with moderate dispersion of individual measurement differences (see Figure 9).

Previous fetal biometry reproducibility studies have reported interobserver agreement limits for fetal head circumference measurements of approximately 3–6% or 12–16 mm (Napolitano et al., 2016; Sarris et al., 2012). Although the agreement limits observed in this study remain broader than typical expert interobserver variability, the achieved performance approaches clinically reported reproducibility ranges for fetal head measurements. In a practical clinical workflow, the system could automatically generate an initial head circumference measurement immediately following image acquisition, which could subsequently be reviewed and refined by the radiologist prior to final reporting. Such a workflow may reduce manual measurement burden and improve efficiency while maintaining clinician oversight in diagnostically important cases.

### 3.4 Multi-structure segmentation model results

The proposed Tri-Attention U-Net outperformed other model architectures for both brain parenchyma and extraaxial CSF segmentation, achieving a mean DSC of 91.28 and 77.38%, respectively. Tables 4, 5 summarize the 5-fold cross validation results for each structure. Overall, all evaluated architectures achieved high performance on the brain parenchyma segmentation task, with relatively small differences across models. In contrast, performance on extraaxial CSF segmentation exhibited greater variability across architectures.

**Table 4:** 5-fold cross validation results for comparative analysis for brain parenchyma class.

Model	DSC	Iou	Sensitivity	Specificity	Precision	hd95	Volume difference
U-Net	90.59% ± 0.58	83.02% ± 0.95	91.65% ± 0.82	98.18% ± 0.02	90.32% ± 0.15	3.71 ± 0.14	6.34% ± 1.83
Attention U-Net	90.47% ± 0.09	82.88% ± 0.15	91.59% ± 0.90	98.13% ± 0.41	90.17% ± 1.99	3.76 ± 0.58	8.16% ± 1.08*
Segresnet	91.02% ± 0.53	83.67% ± 0.09	91.82% ± 0.07	98.26% ± 0.02	90.75% ± 0.11	**3.28 ± 0.14**	**5.09% ± 1.23**
Dynamic U-Net	90.70% ± 0.08	83.27% ± 0.12	**91.93% ± 0.07**	98.18% ± 0.02	90.34% ± 0.15	4.14 ± 0.85	6.35% ± 1.44
Tri-Attention U-Net	**91.28% ± 0.07**	**84.16% ± 0.10**	91.85% ± 0.10	**98.38% ± 0.02**	**91.34% ± 0.14**	3.42 ± 0.70	5.51% ± 1.00

The best results are shown in bold. “*” Indicates statistically significant (*p* < 0.0125). A table including specific *p*-values, 95% confidence interval differences and Cohen’s d effect sizes can be viewed in Supplementary Table 3.

**Table 5:** 5-fold cross validation results for comparative analysis for extraaxial CSF class.

Model	DSC	Iou	Sensitivity	Specificity	Precision	hd95	Volume difference
U-Net	75.54% ± 0.10*	61.18% ± 0.13*	80.35% ± 0.13	97.13% ± 0.13	72.50% ± 0.18	4.69 ± 1.17	18.64% ± 5.34
Attention U-Net	75.88% ± 0.17	61.67% ± 0.21	80.09% ± 0.21	97.28% ± 0.28	73.46% ± 0.16	5.43 ± 1.47	17.61% ± 4.54
Segresnet	76.46% ± 0.10	62.29% ± 0.14*	**81.33% ± 0.21**	97.22% ± 0.03	73.35% ± 0.25	4.33 ± 0.77	19.33% ± 4.90
Dynamic U-Net	76.79% ± 0.1	62.71% ± 0.13	79.86% ± 0.10	**97.52% ± 0.03**	75.17% ± 0.18	**4.09 ± 0.94**	**14.43% ± 3.51**
Tri-Attention U-Net	**77.38% ± 0.08**	**63.46% ± 0.10**	80.71% ± 0.14	**97.52% ± 0.03**	**75.31% ± 0.16**	4.40 ± 0.97	16.59% ± 2.98

The best results are shown in bold. “*” Indicates statistically significant (*p* < 0.0125). A table including specific *p*-values, 95% confidence interval differences and Cohen’s d effect sizes can be viewed in Supplementary Table 4.

All models achieved high DSC values (ranging from 90–91%), indicating strong overlap between predicted and ground truth segmentation results. The Tri-Attention U-Net achieved the highest DSC (91.28% ± 0.07) and IoU (84.16% ± 0.10), while maintaining competitive sensitivity, specificity, and precision. Although the performance differences between models were statistically insignificant, the proposed model consistently outperformed other architectures. The only statistically significant difference observed was in the volume difference metric, where Attention U-Net displayed higher volumetric error compared to the other architectures. This finding may reflect the architecture’s sensitivity to skip-connection feature alignment in the absence of explicit channel recalibration.

Furthermore, SegResNet achieved a slightly lower mean volume difference (5.09%) compared to the proposed model, however, this did not translate into improved overlap-based metrics, as reflected by lower DSC and IoU values, suggesting the difference is not clinically meaningful.

The Tri-Attention U-Net achieved the highest performance across most metrics, including DSC (77.38% ± 0.08) and IoU (63.46% ± 0.10), indicating improved delineation of the extraaxial CSF. Dynamic U-Net achieved the lowest volume difference (14.43% ± 3.51), while the proposed model maintained a competitive volumetric error (16.59% ± 2.98) along with other superior evaluation metrics.

As observed in the brain parenchyma task, only a limited number of comparisons reached statistical significance, primarily in DSC and IoU of U-Net and Attention U-Net.

The comparable performance across models in the second stage (brain parenchyma and extraaxial CSF segmentation) is likely attributable to the constrained region of interest following head segmentation, which simplifies the search space. However, this does not imply reduced task difficulty, as the segmentation of structures such as extraaxial CSF remains challenging, evidenced by its lower DSC. The extraaxial CSF task remains more challenging due to its thin boundaries and decreased contrast in certain volumes. The improved performance of the Tri-Attention U-Net in this task highlights the effectiveness of its attention mechanisms for the SSFP fetal MRIs.

The networks generally achieve accurate segmentation of the brain parenchyma, with minimal errors observed across most cases. However, for earlier gestational ages (for example, 21 weeks), there is a tendency towards slight over-segmentation of the brain parenchyma. In these cases, some models incorrectly label ventricular regions or areas outside the cranial boundary as brain tissue. In contrast, segmentation performance improves at later gestational ages, likely due to increased brain maturation and clearer structural definition.

Segmentation of the extraaxial CSF remains a more challenging task. Most networks commonly over-segment and frequently misclassify internal CSF spaces, such as the ventricles, as extraaxial CSF. This is expected, as both regions contain cerebrospinal fluid and thus share similar intensity characteristics in MRI. In the 35-week gestational age fetus, the Tri-Attention U-Net demonstrates improved delineation of extraaxial CSF, avoiding over-segmentation observed in the Dynamic U-Net, despite their otherwise comparable quantitative performance.

Overall, the Tri-Attention U-Net maintains strong segmentation performance across a broad range of gestational ages, despite the dataset being skewed towards younger fetuses. This suggests that the proposed model is robust to anatomical variability in fetal brain development.

### 3.5 nnU-Net evaluation

The nnU-Net framework was evaluated using the standard configuration without any architectural or training modifications. Two variants were compared: the default nnU-Net implementation based on a conventional U-Net backbone and a modified version incorporating the proposed Tri-Attention U-Net architecture.

Table 6 summarizes the quantitative results for the head segmentation task. Both models achieved strong performance across all evaluation metrics, demonstrating the robustness of the nnU-Net pipeline.

**Table 6:** Results of nnU-Net using the base U-Net and with the Tri-Attention U-Net as the architecture for the head segmentation task.

Model	DSC	Iou	Sensitivity	Specificity	Precision	hd95
Base nnU-Net	93.43% ± 2.45	88.84% ± 2.41	92.13% ± 4.11	**99.58% ± 0.008**	95.41% ± 0.67	**4.17 ± 2.76**
Tri-Attention nnU-Net	**94.48% ± 0.71**	**89.75% ± 1.15**	**93.83% ± 2.26**	99.58% ± 0.01	**95.67% ± 0.90**	5.13 ± 3.63

Best results are shown in bold.

The Tri-Attention nnU-Net achieved a higher DSC (94.48% ± 0.71), IoU (89.75% ± 1.15), sensitivity (93.83% ± 2.26) and precision (95.67% ± 0.90) indicating improved overlap and detection of the head region in whole body fetal MRIs.

However, the Tri-Attention nnU-Net exhibited a slightly higher HD95 (5.13 ± 3.63) compared to the base nnU-Net (4.17 ± 2.76), suggesting marginally less accurate boundary delineation in some cases. Overall, these results indicate that integrating the Tri-Attention mechanism within the nnU-Net framework leads to improved segmentation results while maintaining competitive boundary delineation.

**Figure 10:**
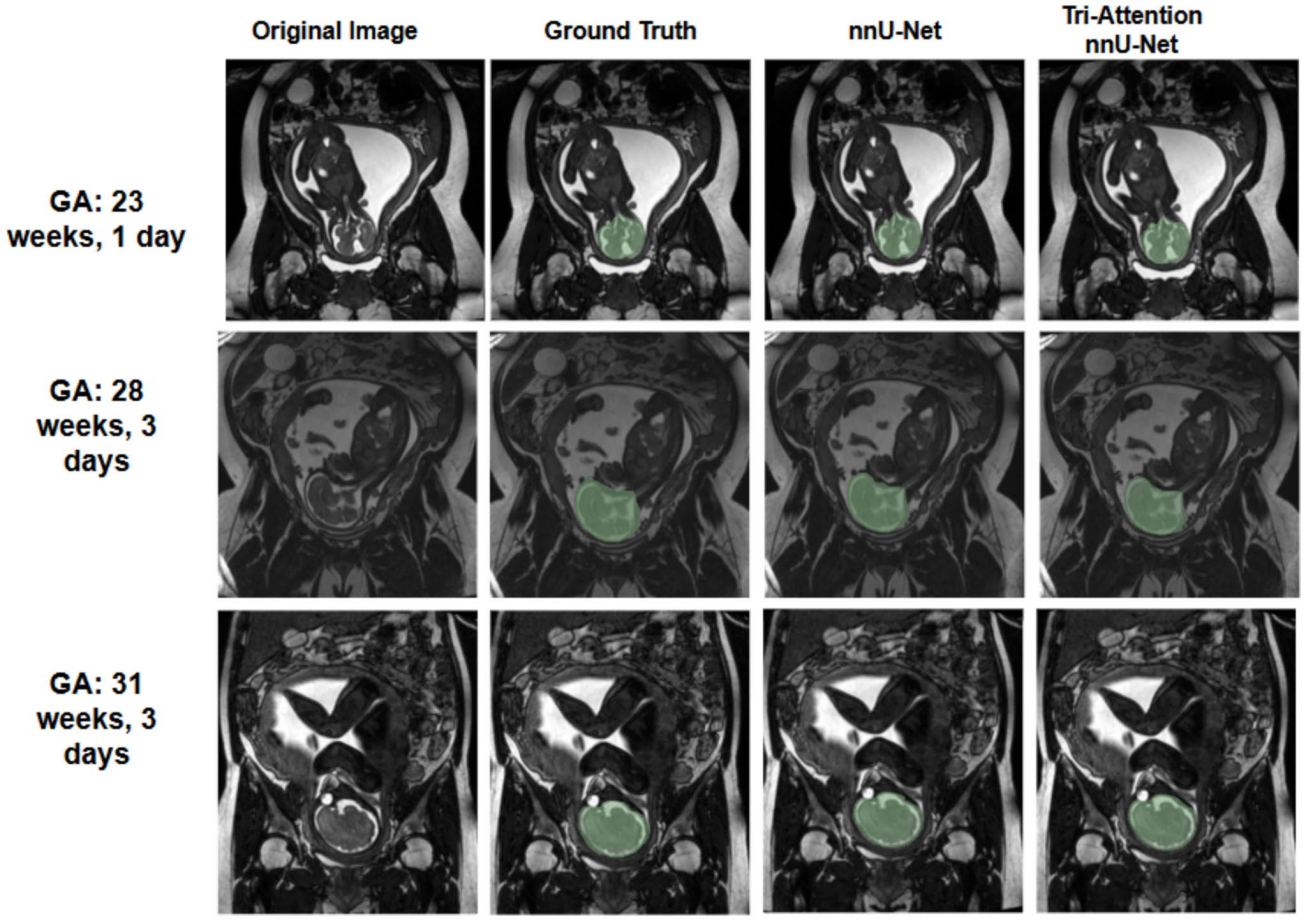
Example outputs from the nnU-Net and the Tri-Attention nnU-Net. The first column displays the whole-body MR image. The second column displays the corresponding ground truth segmentation overlaid on the input. The following columns display prediction segmentation masks for nnU-Net and Tri-Attention nnU-Net, respectively. The green represents the head segmentation mask.

As seen in Figure 10, there is not a significant visual difference between the nnU-Net and the Tri-Attention nnU-Net. This observation is consistent with the quantitative results, where both models achieve similar performance across evaluation metrics. Minor differences are observed at the boundaries, but these are subtle and do not significantly impact the overall segmentation quality.

Both nnU-Net-based models outperform the standalone Tri-Attention U-Net, highlighting the strength of the nnU-Net framework.

Furthermore, the same experiment was completed on the multi-structure segmentation task shown in Tables 7, 8. The base nnU-Net achieved a slightly higher DSC than the Tri-Attention nnU-Net for brain parenchyma segmentation, with a marginal difference of 0.12%. Overall, both models demonstrated highly comparable performance across all evaluation metrics. These results suggest that incorporating the Tri-Attention mechanism does not yield a significant performance gain for brain parenchyma segmentation.

**Table 7:** Results of nnU-Net using the base U-Net and with the Tri-Attention U-Net as the architecture for the multi-structure segmentation task.

Model	DSC	Iou	Sensitivity	Specificity	Precision	hd95	Volume difference
Base nnU-Net	**93.70% ± 0.04**	**88.26% ± 0.11**	**94.79% ± 0.08**	98.67% ± 0.02	92.75% ± 0.11	**1.50 ± 0.10**	**3.71% 1.52**
Tri-Attention nnU-Net	93.58% ± 0.07	88.04% ± 0.12	94.40% ± 0.08	**98.70% ± 0.02**	**92.88% ± 0.09**	1.57 ± 0.13	3.68% ± 1.33

This table shows the results for the first class, the brain parenchyma. Best results are shown in bold.

**Table 8:** Results of nnU-Net using the base U-Net and with the Tri-Attention U-Net as the architecture for the multi-structure segmentation task.

Model	DSC	Iou	Sensitivity	Specificity	Precision	hd95	Volume difference
Base nnU-Net	**82.75% ± 0.01**	**70.94% ± 0.19**	**84.00% ± 0.17**	**98.33% ± 0.03**	**82.38% ± 0.31**	**1.89 ± 0.38**	**11.51% ± 4.37**
Tri-Attention nnU-Net	82.49% ± 0.15	70.57% ± 0.20	83.84% ± 0.22	98.30% ± 0.03	82.17% ± 0.32	1.98 ± 0.43	12.52% ± 4.39

This table shows the results for the second class, the extraaxial CSF. Best results are shown in bold.

The extraaxial CSF segmentation results in Table 8 follow a similar trend to those observed for brain parenchyma segmentation. The base nnU-Net slightly outperforms the Tri-Attention nnU-Net across all metrics, although the differences are marginal. Both models exhibit lower performance compared to the brain parenchyma task, which is expected given the increased difficulty of extraaxial CSF segmentation due to its low contrast boundaries and higher susceptibility to partial volume effects in fetal MRI.

Despite the inclusion of attention mechanisms, the Tri-Attention nnU-Net does not demonstrate a meaningful advantage and may introduce minor inconsistencies in some cases. Nevertheless, both nnU-Net-based approaches outperform the standalone Tri-Attention U-Net, reinforcing the robustness and generalizability of the nnU-Net framework for challenging segmentation tasks.

**Figure 11:**
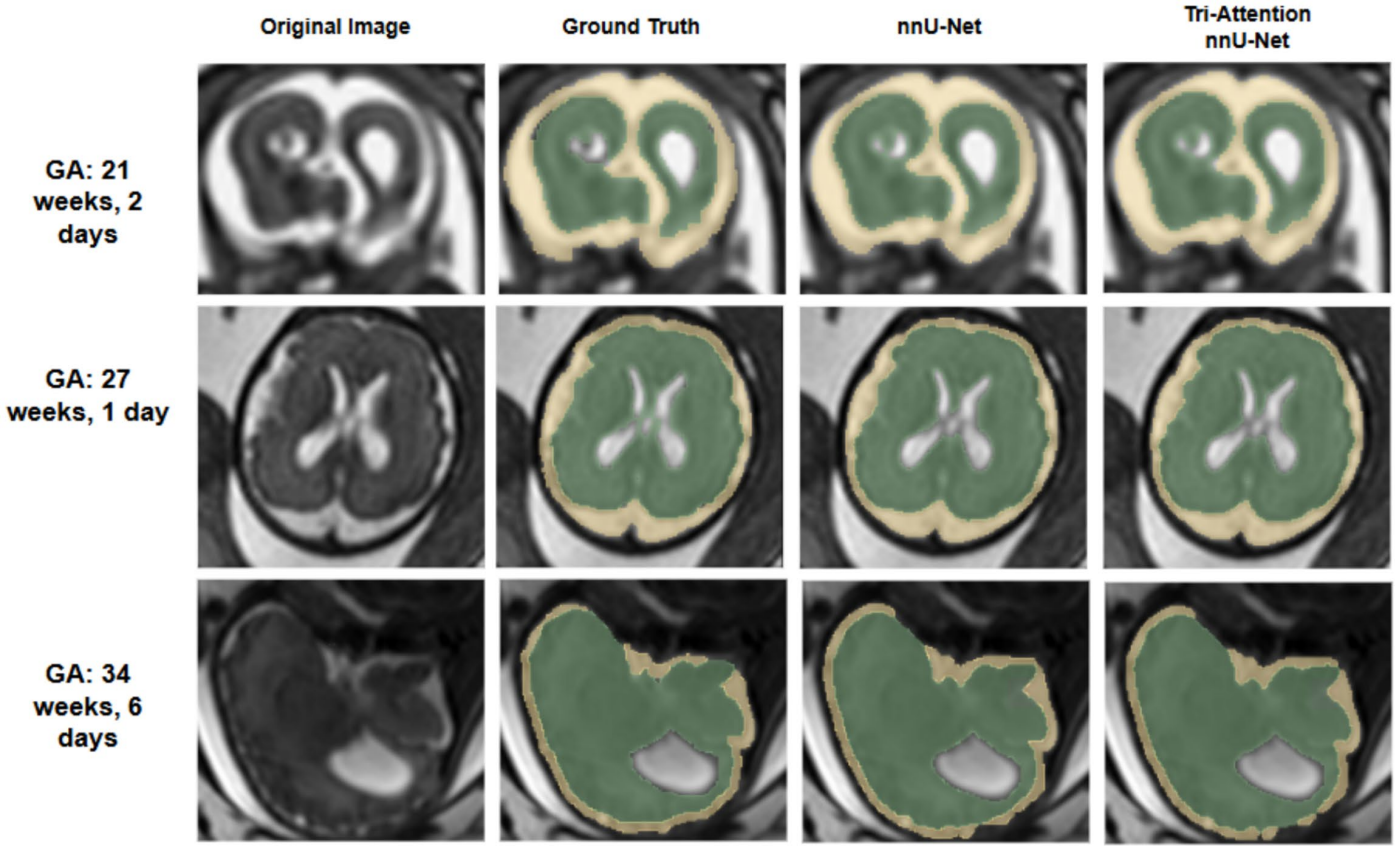
Example outputs from the nnU-Net and the Tri-Attention nnU-Net. The first column displays the whole body MR image. The second column displays the corresponding ground truth segmentation overlaid on the input. The following columns display prediction segmentation masks for nnU-Net and Tri-Attention nnU-Net, respectively. The green and yellow highlights represent the brain parenchyma and extraaxial CSF, respectively.

There are minor differences observed in the multi-structure segmentation results shown in Figure 11. In the 27-week gestational age case, both nnU-Net and Tri-Attention nnU-Net slightly under segment the brain parenchyma near the lateral ventricles. In the 34-week gestational age case, partial volume effects reduce the visibility of the extraaxial CSF, resulting in segmentation challenges for both models.

### 3.6 Failure case analysis

**Figure 12:**
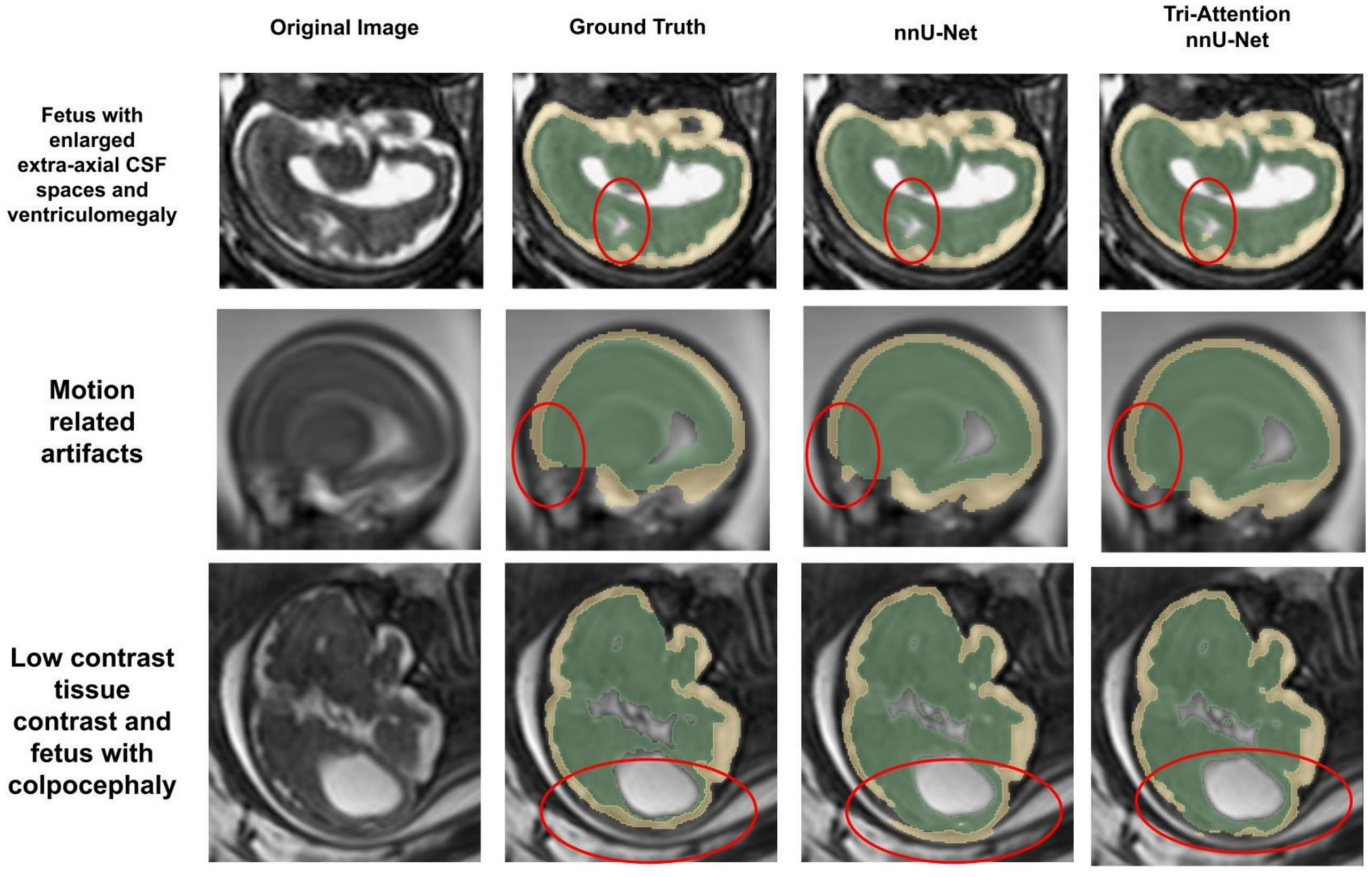
Example failure case outputs from the nnU-Net and the Tri-Attention nnU-Net. The first column displays the whole body MR image. The second column displays the corresponding ground truth segmentation overlaid on the input. The following columns display prediction segmentation masks for nnU-Net and Tri-Attention nnU-Net, respectively. The green and yellow highlights represent the brain parenchyma and extraaxial CSF, respectively. The red circles show regions where over-segmentation or under-segmentation occurs.

Representative failure cases for the multi-structure segmentation task are shown in Figure 12. The primary failure modes were associated with fetuses with common abnormalities in our dataset (such as enlarged extraaxial CSF spaces, ventriculomegaly and colpocephaly), motion related artifacts and low contrast tissue contrast.

In the fetus with enlarged extraaxial CSF and ventriculomegaly, there is slight over-segmentation within the gyrus region highlighted by the red circle. The effect is more pronounced in the Tri-Attention nnU-Net, potentially because the additional attention mechanisms increase sensitivity to ambiguous anatomical regions, resulting in false positive predictions. Furthermore, for the fetus affected by motion related artifacts, the selected example exhibits moderate motion that reduces visibility of the boundaries between the extraaxial CSF and brain parenchyma. However, the Tri-Attention nnU-Net outperforms the baseline nnU-Net, as it successfully captures the extraaxial CSF near the occipital lobe which is partially missed by the nnU-Net. Lastly, an image with low contrast tissue contrast and colpocephaly was evaluated. In this case, the nnU-Net incorrectly segments both the brain parenchyma and the extraaxial CSF, as seen in the red circle, likely due to the poor contrast between these tissues. As a result, the model misclassifies adjacent amniotic fluid outside the head as extraaxial CSF. The Tri-Attention nnU-Net demonstrates a similar pattern for the brain parenchyma, but rather than misclassifying the surrounding amniotic fluid, it fails to segment the extraaxial CSF in the highlighted region and omits this structure entirely.

It was found that attention mechanisms improved performance in head localization but provided limited benefit for fine-grained segmentation tasks like multi-structure segmentation. This may be attributed to the way attention gates learn soft spatial masks at the skip-connection level, suppressing feature activations originating from regions considered irrelevant by the gating signal. This mechanism is particularly effective for the head localization task, where the main challenge is distinguishing the fetal head from anatomically similar structures, such as the fetal body and maternal tissues, across a large field of view. In this setting, the gating attention acts as a coarse spatial filtering mechanism that suppresses irrelevant activations before they are propagated through the decoder, resulting in improved DSC and sensitivity.

On the other hand, fine segmentation of the brain parenchyma and extraaxial CSF represents a boundary-sensitive task performed within an already localized and cropped region of interest. At this stage, the primary challenge shifts from global localization to accurate delineation of subtle anatomical boundaries, specifically along the cortical surface and at the interfaces between ventricular and extraaxial CSF. Attention gates are not specifically designed to enhance local boundary precision, as they generate spatially smooth weighting maps derived from low frequency contextual features. Consequently, attention gates are less effective at refining boundary transitions required for precise contour delineation. This behaviour is reflected in the HD95 results presented in Tables 6, 7, where the Tri-Attention nnU-Net consistently demonstrates higher HD95 values (indicating poorer boundary delineation) than the baseline nnU-Net for the multi-structure segmentation tasks, despite achieving comparable DSC. These findings suggest that attention gating primarily benefits coarse localization rather than the refinement of complex anatomical boundaries.

The limited dataset size might have also contributed to a reduced generalization performance of the Tri-Attention architecture. Relative to the baseline nnU-Net, the Tri-Attention architecture introduces additional learnable parameters through the incorporation of each attention module. Due to the relatively small dataset size, this could have led to overfitting. This interpretation is supported by the increased variability observed for the Tri-Attention nnU-Net across several evaluation metrics. For example, the standard deviation of the DSC for extraaxial CSF segmentation increased relative to the baseline nnU-Net (0.15 vs. 0.01 as seen in Table 8), indicating reduced consistency in generalization across folds. Such elevated variability is often indicative of overfitting, suggesting that the model may have become more sensitive to fold-specific characterisitics rather than learning features that generalize robustly across the dataset.

Some of the incorporated attention mechanisms may also provide functionality that overlaps with existing nnU-Net design features. In particular, the patch-based training strategy with random spatial sampling implicitly performs spatial weighting during optimization. Consequently, adding multiple attention components, such as ECA, ASPP, SE and attention gates, may introduce redundant representational capacity and increased complexity without performance gains. This effect can be seen in the brain parenchyma segmentation task, where performance is already satisfactory, so the attention modules provides little to no measurable benefit. In contrast, tasks that rely more heavily on global spatial context, such as the head localization task, are less susceptible to this redundancy and may benefit more from the additional attention mechanisms. The reduced performance gains of the Tri-Attention nnU-Net on multi-structure segmentation tasks, where the region of interest is already spatially constrained, supports this interpretation.

These findings demonstrate the feasibility of an accurate, fully automated fetal brain biometry from SSFP MRI with the potential for clinical integration. The final pipeline incorporates a Tri-Attention nnU-Net for head segmentation and a baseline nnU-Net for multi-structure segmentation. The entire pipeline (including head segmentation, head circumference extraction and multi-structure segmentation) achieved an average inference time of 48.07 ± 13.78 s per volume on an NVIDIA GeForce RTX 4090 (24GB) GPU, with a peak memory usage of approximately 7.82 GB.

### 3.7 Limitations

There are several limitations in this study. Firstly, the dataset originates from a single center, which may limit the generalizability of the model to fetal MRIs acquired at other institutions. Additionally, the pipeline was trained on a single MRI sequence type that is primarily used only in research settings, which may further restrict clinical applicability. Although the ground truth masks were validated by an expert, the segmentations were performed in-house, which may introduce some errors. Ground truth segmentations were verified by a single expert, which means no inter−/intra-observer comparison could be completed. Future work should aim to incorporate multiple experts and annotators to assess ground truth uncertainty. Secondly, the performance of the overall pipeline depends on the accuracy of the head segmentation model, as both the head circumference estimation and multi-structure segmentation stages rely on accurate head localization and cropping. Lastly, the multi-structure segmentation task was limited to two classes due to the time-intensive nature of manual segmentation. Future work should aim to include additional structures, such as the ventricular system and individual brain structures, to enable more comprehensive fetal brain analysis.

## 4 Conclusion

This study presents a fully automated deep learning pipeline for fetal brain segmentation and biometric analysis using 3D SSFP MRI. The proposed framework leverages nnU-Net for robust segmentation with a Tri-Attention nnU-Net variant improving head localization, while the baseline nnU-Net provides more consistent performance for multi-structure segmentation. These findings highlight the importance of task-specific model selection, where attention mechanisms benefit global localization tasks but may not generalize to finer anatomical structures.

By leveraging whole-body SSFP acquisitions, the proposed pipeline eliminates the need for super-resolution reconstruction and directly provides volumetric measurements of key fetal brain biomarkers, such as head circumference, brain parenchyma, and extraaxial CSF volumes. Furthermore, the integration of a PCA-based reorientation algorithm ensures consistent anatomical alignment and enables automatic head circumference estimation in the true axial plane.

In conclusion, this work demonstrates the feasibility of accurate, fully automated fetal brain biometry from SSFP MRI and highlights the robustness and adaptability of nnU-Net-based approaches for complex fetal MRI segmentation tasks. Future work will focus on validation across larger and more diverse clinical datasets, as well as expanding the range of segmented structures and derived biometric measurements.

## Data Availability

The data analyzed in this study is subject to the following licenses/restrictions: the datasets generated during and/or analyzed during the current study are not publicly available due to legal restrictions but are available from the corresponding author on reasonable request of a data sharing agreement. The code will be made available when published at: https://github.com/DrSussmanLab/fetal-brain-seg. Requests to access these datasets should be directed to Dafna Sussman, dafna.sussman@torontomu.ca.
