# Supplementary material for: An end-to-end pipeline for automated fetal brain segmentation and biometry from 3D SSFP MRI
Source: Front Neurosci. 2026 Jul 9;20:1870124. doi: 10.3389/fnins.2026.1870124 (PMC13391945; doi:10.3389/fnins.2026.1870124)
Supplement: Supplementary file 1 [file Table_1.DOCX]

Table 1: 5-Fold cross validation results for comparative analysis on head segmentation dataset. The best results are shown in bold. P values are shown in brackets (denoted with a ‘p’), along with 95% confidence interval differences (denoted with a ‘CI’) and the Cohen’s d effect sizes for each evaluation metric (denoted with a ‘d’).

| Model | DSC | Iou | Sensitivity | specificity | precision | hd95 |
| --- | --- | --- | --- | --- | --- | --- |
| U-Net | 80.43% ± 2.86  (p=0.019, CI= [1.43, 9.12], d=1.70) | 68.79%± 3.59  (p=0.0259, CI= [1.38, 12.64], d=1.5464) | 94.88% ±2.41  (p=0.031, CI= [0.47, 5.75], d=1.4610) | 99.64%± 0.09  (p=0.0646, CI= [-0.01, 0.21], d=1.1316) | 72.03% ±5.63  (p=0.0401, CI= [0.48, 12.75], d=1.3394) | 24.15 ± 7.45  (p=0.099, CI= [-24.92, 3.26], d=-0.9544) |
| attention u-net | 82.31%± 4.25  (p=0.051, CI= [-0.02, 6.79], d=1.23) | 71.32%± 5.37  (p=0.0552, CI= [-0.16, 9.13], d=1.1986) | 95.47%± 2.71  (p=0.0719, CI= [-0.36, 5.39], d=1.0873) | 99.69%± 0.10  (p=0.2003, CI= [-0.04, 0.15], d=0.6851) | 75.04%± 7.09  (p=0.1613, CI= [-2.23, 9.44], d=0.7674) | 23.72 ±14.07  (p=0.026, CI= [-18.79, -2.01], d=-1.53) |
| segresnet | 77.73% ± 5.40*  (p=0.01, CI= [3.18, 12.76], d=2.07) | 64.68%± 6.80*  (p=0.0081, CI= [4.81, 17.44], d=2.1879) | 95.88% ± 2.63  (p=0.1233, CI= [-0.89, 5.10], d=0.8711) | 99.56%± 0.09*  (p=0.0116, CI= [0.07, 0.29], d=1.9712) | 68.22% ± 5.99*  (p=0.0084, CI= [4.45, 16.41], d=2.1656) | **9.30± 3.07**  (p=0.323, CI= [-5.87, 13.89], d=0.5042) |
| dynamic u-net | 82.51% ± 5.19  (p=0.21, CI= [-2.80, 9.17], d=0.66) | 71.52%± 6.92  (p=0.2343, CI= [-4.22, 12.79], d=0.6258) | 96.11%± 1.88  (p=0.0383, CI= [0.16, 3.58], d=1.3603) | 99.68%± 0.12  (p=0.2683, CI= [-0.08, 0.21], d=0.5744) | 74.76%± 6.92  (p=0.2863, CI= [-4.89, 12.65], d=0.5499) | 14.71± 1.45  (p=0.6874, CI= [-10.33, 7.54], d=-0.1936) |
| tri-attention u-net | **85.70% ± 3.38** | **75.79%± 4.67** | **97.98% ± 0.53** | **99.74%± 0.05** | **78.65%± 4.05** | 13.32 ± 7.67 |

Table 2: 5-Fold cross validation results for ablation study on head segmentation dataset. The best results are shown in bold. Pairwise comparisons were conducted between the full Tri-Attention U-Net and each ablation variant where ‘*’ indicates statistical significance (p<0.01 due to 5 comparisons with Bonferroni-correction). The best results are shown in bold. P values are shown in brackets (denoted with a ‘p’), along with 95% confidence interval differences (denoted with a ‘CI’) and the Cohen’s d effect sizes for each evaluation metric (denoted with a ‘d’).

| Model | DSC | Iou | Sensitivity | specificity | precision | hd95 |
| --- | --- | --- | --- | --- | --- | --- |
| baseline u-net | 80.43% ± 2.86  (p=0.019, CI= [1.43, 9.12], d=1.70) | 68.79%± 3.59  (p=0.0259, CI= [1.38, 12.64], d=1.5464) | 94.88% ±2.41  (p=0.031, CI= [0.47, 5.75], d=1.4610) | 99.64%± 0.09  (p=0.0646, CI= [-0.01, 0.21], d=1.1316) | 72.03% ±5.63  (p=0.0401, CI= [0.48, 12.75], d=1.3394) | 24.15 ± 7.45  (p=0.099, CI= [-24.92, 3.26], d=-0.9544) |
| eca only | 74.47%± 3.22*  (p=0.0032, CI= [6.31, 16.15], d=2.8364) | 60.89%± 3.83*  (p=0.0021, CI= [9.08, 20.75], d=3.1728) | 96.02%± 1.92  (p=0.0861, CI= [-0.44, 4.39], d=1.0133) | 99.46%± 0.07*  (p=0.0011, CI= [0.19, 0.37], d=3.7326) | 63.65%± 3.55*  (p=0.0020, CI= [9.19, 20.80], d=3.2070) | 16.20 ±4.21  (p=0.3828, CI= [-11.05, 5.28], d=-0.4380) |
| aspp only | 72.02% ± 5.16*  (p=0.0025, CI= [8.07, 19.30], d=3.0250) | 57.91%± 6.55*  (p=0.0022, CI= [10.80, 24.99], d=3.1307) | 95.73% ± 1.94  (p=0.1153, CI= [-0.87, 5.38], d=0.8971) | 99.39%± 0.15*  (p=0.0074, CI= [0.16, 0.55], d=2.2417) | 60.69% ± 7.24*  (p=0.0044, CI= [9.37, 26.55], d=2.5967) | 19.29± 4.15  (p=0.2084, CI= [-17.05, 5.09], d=-0.6701) |
| attention gate only | 75.87% ± 4.24  (p=0.0143, CI= [3.25, 16.42], d=1.8541) | 62.61%± 13.45  (p=0.0117, CI= [4.80, 21.18], d=1.9687) | 95.73%± 1.92  (p=0.0633, CI= [-0.20, 4.72], d=1.1404) | 99.51%± 0.11  (p=0.0260, CI= [0.04, 0.41], d=1.5430) | 65.98%± 5.84  (p=0.0153, CI= [4.02, 21.32], d=1.8182) | 15.20± 4.21  (p=0.7043, CI= [-14.69, 10.92], d=-0.1824) |
| se only | 77.72% ± 2.84*  (p=0.0055, CI= [3.90, 12.05], d=2.4327) | 64.88%± 3.40*  (p=0.0031, CI= [6.18, 15.66], d=2.8622) | 95.98%± 2.10  (p=0.1251, CI= [-0.87, 4.90], d=0.8653) | 99.56%± 0.07*  (p=0.0074, CI= [0.08, 0.28], d=2.2400) | 67.99%± 4.21*  (p=0.0104, CI= [4.16, 17.15], d=2.0376) | **11.25± 5.82**  (p=0.4942, CI= [-5.56, 9.69], d=0.3360) |
| tri-attention u-net | **85.70% ± 3.38** | **75.79%± 4.67** | **97.98% ± 0.53** | **99.74%± 0.05** | **78.65%± 4.05** | 13.32 ± 7.67 |

Table 3: 5-Fold cross validation results for comparative analysis for brain parenchyma class. The best results are shown in bold. P values are shown in brackets (denoted with a ‘p’), along with 95% confidence interval differences (denoted with a ‘CI’) and the Cohen’s d effect sizes for each evaluation metric (denoted with a ‘d’).

| Model | DSC | Iou | Sensitivity | specificity | precision | hd95 | Volume Difference |
| --- | --- | --- | --- | --- | --- | --- | --- |
| U-Net | 90.59% ± 0.58  (p=0.0792, CI= [-1.50, 0.13], d=-1.047) | 83.02%± 0.95  (p=0.0584, CI= [-2.35, 0.07], d=-1.175) | 91.65% ± 0.82  (p=0.5301, CI= [-1.05, 0.63], d=-0.307) | 98.18%± 0.02  (p=0.0898, CI= [-0.45, 0.05], d=-0.996) | 90.32% ± 0.15  (p=0.0848, CI= [-2.26, 0.22], d=-1.020) | 3.71± 0.14  (p=0.4078, CI= [-0.59, 1.18], d=0.413) | 6.34% ± 1.83  (p=0.4295, CI= [-1.79, 3.45], d=0.393) |
| attention u-net | 90.47% ± 0.09  (p=0.0207, CI= [-1.42, -0.20], d=-1.659) | 82.88% ± 0.15  (p=0.0203, CI= [-2.23, -0.33], d=-1.669) | 91.59%± 0.90  (p=0.5163, CI= [-1.30, 0.77], d=-0.318) | 98.13%± 0.41  (p=0.1315, CI= [-0.62, 0.12], d=-0.846) | 90.17% ± 1.99  (p=0.1031, CI= [-2.73, 0.37], d=-0.941) | 3.76 ± 0.58  (p=0.1764, CI= [-0.23, 0.92], d=0.733) | 8.16% ± 1.08*  (p=0.0100, CI= [1.05, 4.24], d=2.058) |
| segresnet | 91.02%± 0.53  (p=0.3962, CI= [-1.01, 0.50], d=-0.425) | 83.67%± 0.09  (p=0.2912, CI= [-1.63, 0.64], d=-0.543) | 91.82%± 0.07  (p=0.9503, CI= [-1.67, 1.60], d=-0.030) | 98.26%± 0.02  (p=0.0889, CI= [-0.28, 0.03], d=-1.000) | 90.75% ± 0.11  (p=0.0592, CI= [-1.21, 0.04], d=-1.169) | **3.28± 0.14**  (p=0.7396, CI= [-1.16, 0.89], d=-0.159) | **5.09% ± 1.23**  (p=0.6244, CI= [-2.62, 1.78], d=0.237) |
| dynamic u-net | 90.70% ± 0.08  (p=0.0898, CI=[-1.30, 0.14], d=-0.996) | 83.27%± 0.12  (p=0.0929, CI= [-2.03, 0.24], d=-0.983) | **91.93%± 0.07**  (p=0.8333, CI= [-0.88, 1.03], d=-0.030) | 98.18% ± 0.02  (p=0.0158, CI= [-0.35, -0.06], d=-1.801) | 90.34%± 0.15  (p=0.0156, CI= [-1.69, -0.31], d=-1.806) | 4.14± 0.85  (p=0.2237, CI= [-0.6784, 2.1372], d=0.643) | 6.35% ± 1.44  (p=0.3618, CI= [-1.42, 3.09], d=0.460) |
| tri-attention u-net | **91.28% ± 0.07** | **84.16% ± 0.10** | 91.85% ± 0.10 | **98.38%± 0.02** | **91.34% ± 0.14** | 3.42 ± 0.70 | 5.51% ± 1.00 |

Table 4: 5-Fold cross validation results for comparative analysis for extraaxial CSF class. The best results are shown in bold. P values are shown in brackets (denoted with a ‘p’), along with 95% confidence interval differences (denoted with a ‘CI’) and the Cohen’s d effect sizes for each evaluation metric (denoted with a ‘d’).

| Model | DSC | Iou | Sensitivity | specificity | precision | hd95 | Volume Difference |
| --- | --- | --- | --- | --- | --- | --- | --- |
| U-Net | 75.54%± 0.10*  (p=0.0011, CI= [-2.45, -1.23], d=-3.720) | 61.18%± 0.13*  (p=0.0009, CI= [-3.00, -1.57], d=-3.971) | 80.35%± 0.13  (p=0.7553, CI= [-3.38, 2.66], d=-0.149) | 97.13% ± 0.13  (p=0.0971, CI= [-0.88, 0.11], d=-0.965) | 72.50% ± 0.18  (p=0.0507, CI= [-5.63, 0.01], d=-1.236) | 4.69± 1.17  (p=0.4629, CI= [-0.70, 1.27], d=0.363) | 18.64% ± 5.34  (p=0.3479, CI= [-3.30, 7.40], d=0.475) |
| attention u-net | 75.88% ± 0.17  (p=0.0425, CI= [-2.91, -0.08], d=-1.313) | 61.67% ± 0.21  (p=0.0457, CI= [-3.53, -0.06], d=-1.281) | 80.09%± 0.21  (p=0.5644, CI= [-3.34, 2.11], d=-0.281) | 97.28% ± 0.28  (p=0.1350, CI= [-0.58, 0.11], d=-0.836) | 73.46%± 0.16  (p=0.0767, CI= [-4.01, 0.32], d=-1.061) | 5.43 ± 1.47  (p=0.1645, CI= [-0.65, 2.71], d=0.760) | 17.61%± 4.54  (p=0.5459, CI= [-3.27, 5.31], d=0.295) |
| segresnet | 76.46% ± 0.10  (p=0.0125, CI= [-1.50, -0.33], d=-1.929) | 62.29%± 0.14*  (p=0.0106, CI= [-1.89, -0.45], d=-2.022) | **81.33% ± 0.21**  (p=0.5430, CI= [-1.97, 3.20], d=0.297) | 97.22%± 0.03  (p=0.1246, CI= [-0.72, 0.13], d=-0.867) | 73.35% ± 0.25  (p=0.0918, CI= [-4.44, 0.51], d=-0.988) | 4.33± 0.77  (p=0.8449, CI= [-0.97, 0.83], d=-0.093) | 19.33%± 4.90  (p=0.3151, CI= [-3.89, 9.38], d=0.513) |
| dynamic u-net | 76.79% ± 0.1  (p=0.0682, CI= [-1.23, 0.07], d=-1.109) | 62.71%± 0.13  (p=0.0511, CI= [-1.50, 0.01], d=-1.232) | 79.86% ± 0.10  (p=0.0429, CI= [-1.65, -0.04], d=-1.309) | **97.52%± 0.03**  (p=0.9219, CI= [-0.11, 0.12], d=0.047) | 75.17%± 0.18  (p=0.6548, CI= [-0.94, 0.66], d=-0.216) | **4.09± 0.94**  (p=0.2559, CI= [-0.94, 0.33], d=-0.592) | **14.43% ± 3.51**  (p=0.0187, CI= [-3.72, -0.59], d=-1.712) |
| tri-attention u-net | **77.38% ± 0.08** | **63.46%± 0.10** | 80.71%± 0.14 | **97.52% ± 0.03** | **75.31%± 0.16** | 4.40 ±0.97 | 16.59%± 2.98 |
